# Co-Infection of Infectious Hypodermal and Hematopoietic Necrosis Virus (IHHNV) and White Spot Syndrome Virus (WSSV) in the Wild Crustaceans of Andaman and Nicobar Archipelago, India

**DOI:** 10.3390/v13071378

**Published:** 2021-07-15

**Authors:** Kandasamy Saravanan, Jayasimhan Praveenraj, Rajendran Kiruba-Sankar, Varsha Devi, Utpal Biswas, Thangaraj Sathish Kumar, Arun Sudhagar, Mansour El-Matbouli, Gokhlesh Kumar

**Affiliations:** 1 Division of Fisheries Science, Indian Council of Agricultural Research-Central Island Agricultural Research Institute, Port Blair 744105, Andaman and Nicobar Islands, India; praveenraj.j@icar.gov.in (J.P.); rkiruba.sankar@icar.gov.in (R.K.-S.); varmaysha21@gmail.com (V.D.); utpalbt22@gmail.com (U.B.); 2 Aquatic Animal Health and Environment Division, Indian Council of Agricultural Research-Central Institute of Brackishwater Aquaculture, 75, Santhome High Road, Chennai 600028, Tamil Nadu, India; sathishkumar.t@icar.gov.in; 3 Peninsular and Marine Fish Genetic Resources Centre, Indian Council of Agricultural Research-National Bureau of Fish Genetic Resources, Ernakulam North P.O., Kochi 682018, Kerala, India; arun.sudhagar@icar.gov.in; 4 Clinical Division of Fish Medicine, University of Veterinary Medicine Vienna, Veterinarplatz 1, 1210 Vienna, Austria; Mansour.El-Matbouli@vetmeduni.ac.at

**Keywords:** IHHNV, WSSV, co-infection, wild crustaceans, disease surveillance

## Abstract

The present study was intended to screen the wild crustaceans for co-infection with Infectious Hypodermal and Hematopoietic Necrosis Virus (IHHNV) and White Spot Syndrome Virus (WSSV) in Andaman and Nicobar Archipelago, India. We screened a total of 607 shrimp and 110 crab samples using a specific polymerase chain reaction, and out of them, 82 shrimps (13.5%) and 5 (4.5%) crabs were found positive for co-infection of IHHNV and WSSV. A higher rate of co-infection was observed in *Penaeus monodon* and *Scylla serrata* than other shrimp and crab species. The nucleotide sequences of IHHNV and WSSV obtained from crab in this present study exhibited very high sequence identity with their counterparts retrieved from various countries. Histopathological analysis of the infected shrimp gill sections further confirmed the eosinophilic intra-nuclear cowdry type A inclusion bodies and basophilic intra-nuclear inclusion bodies characteristics of IHHNV and WSSV infections, respectively. The present study serves as the first report on co-infection of WSSV and IHHNV in Andaman and Nicobar Archipelago, India and accentuates the critical need for continuous monitoring of wild crustaceans and appropriate biosecurity measures for brackishwater aquaculture.

## 1. Introduction

Viruses pose a major threat to human and animal health in comparison to other pathogens across the globe. The rapid growth of the aquaculture sector was stagnated by the incidence of diseases mainly caused by infectious viruses leading to crop losses and economic impacts. Globally, more than 20 viruses have so far been recorded for shrimp and some of these are found to be responsible for epidemics severely impacting its health [1,2]. White Spot Syndrome Virus (WSSV) and Infectious Hypodermal and Hematopoietic Necrosis Virus (IHHNV) are the widespread and most prevalent pathogens causing mass mortality and growth retardation leading to huge economic losses in crustaceans [3,4,5]. WSSV was known to incur losses to the tune of $10 billion in penaeid culture [6,7] whereas IHHNV is known to cause runting syndrome and reduce the market value of penaeid shrimps up to 10% to 50% [8,9]. Due to the worldwide impact, both viruses were enlisted as notifiable crustacean pathogens by the World Organization for Animal Health [10]. IHHNV is the smallest known penaeid virus harbouring a single-stranded DNA [11]. WSSV is the double-stranded DNA virus known to infect more than 40 penaeid and non-penaeid species of crustaceans [11,12]. Both the viruses are known to have a wide host range among which mud crabs are considered as a potential threat to shrimp aquaculture as they are highly tolerant and serve as carriers for a long period without exhibiting any signs and symptoms [13,14].

The incidences of viral diseases in aquaculture species, particularly crustaceans such as shrimps and crabs have been well studied [5,15,16,17,18,19,20,21,22]. On the other hand, studies on viral infections in wild marine crustaceans are restricted to few reports [23,24,25,26,27,28]. It is also reported that once these infections were established it would have an impact on wild crustaceans [29]. Even though co-infections are common and frequently occurring phenomena in wild conditions, not much attention has been paid to the co-infection of aquatic animals as the studies are mainly restricted to single infections [5,30]. Further, co-infections can be caused by heterologous or homologous pathogens and the interaction between the co-infecting pathogens may be either synergistic or antagonistic on the infected host [31,32,33].

Crustaceans are the potential hosts for both WSSV and IHHNV, which may spread through vectors by horizontal transmission [34] and vertical transmission via infected broodstocks. Further, the infected hosts and vectors may assist in introducing or spreading of these viruses into new geographical locations [35]. Although there is no effective treatment to cure the viral infections in crustaceans, biosecurity measures such as avoiding the source of infection, disease-free broodstocks or seeds, maintaining better water quality, augmenting the disease resistance of the host, and hindering the disease transmission process are the major requisites to put the viral infection under control in culture conditions which may not be feasible in endemic areas of these viruses [24,36].

Andaman and Nicobar Archipelago is located in the Bay of Bengal, in close proximity to the Southeast Asian Countries than mainland India. Andaman and Nicobar Archipelago comprises of 572 islands divided into three districts namely, South Andaman, North and Middle Andaman, and Nicobar. At present, brackishwater aquaculture is not practised as a commercial venture in the archipelago, whereas the sector has the potential to develop in the future due to the tremendous economic prospects. Only scanty reports are available on the incidences of diseases in shrimps such as vibriosis, Laem–Singh Virus, WSSV, and IHHNV in Andaman and Nicobar Archipelago [37,38,39,40,41]. However, disease screening of wild aquatic animals is necessary in order to avoid the negative impact of possible futuristic disease outbreaks in brackishwater aquaculture [26]. Keeping in view of the potential of the brackishwater aquaculture sector, the present study reports the co-infection of WSSV and IHHNV for the first time in wild crustaceans such as shrimps and crabs collected from various fish landing centres of Andaman and Nicobar Archipelago, India. Further, the present study also highlights the degree of co-infection among the wild crustaceans across the fish landing centres and accentuates the necessity for proper biosecurity measures before venturing into large-scale brackishwater aquaculture.

## 2. Materials and Methods

### 2.1. Collection of Samples

**Figure 1:**
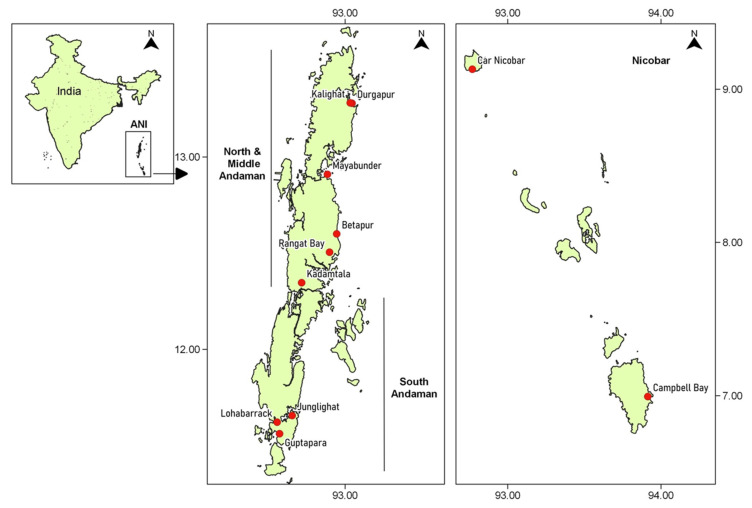
Map showing the sample collection sites in Andaman and Nicobar Archipelago (ANI), India. The wild shrimp and crab samples were collected from various fish landing centres located in all the three districts of ANI namely, North and Middle Andaman, South Andaman and Nicobar districts.

Wild marine shrimp and crab samples were collected from various fish landing centres located at Andaman and Nicobar Islands (Figure 1) from July 2018 to December 2020. Altogether, 607 shrimp samples comprising of *Penaeus monodon, P. merguiensis, P. indicus* and *P. penicillatus* were collected with mean length (cm) and mean weight (g) ranging from 12.7 ± 0.7 to 22.5 ± 0.8 and 14.3 ± 0.9 to 87.0 ± 10.8, respectively. Similarly, 110 crab samples comprising of *Scylla serrata, S. tranquebarica*, *Portunus pelagicus* and *P. reticulatus* were collected with mean carapace length (cm) and mean weight (g) ranging from 4.8 ± 0.2 to 8.4 ± 1.0 and 71.4 ± 6.7 to 350.9 ± 71.2, respectively. Tissues such as pleopod and gill in case of shrimp samples and cheliped muscle and gill in case of crab samples were dissected out and preserved in 90% ethanol for further use. Similarly, the tissue samples were also preserved in Davidson’s fixative for histopathology.

### 2.2. DNA Isolation and PCR Amplification of Target Viruses

DNA was isolated from the tissue samples using DNAzol reagent (Invitrogen, Waltham, MA, USA) by following the manufacturer’s instructions. The extracted DNA was used for the PCR detection of IHHNV and WSSV as per the protocols of Tang et al. [42] and Kimura et al. [43], respectively. Internal positive and negative controls were included in all the reactions. The amplified products were resolved in agarose gel electrophoresis and visualised using a gel documentation system (Bio-Rad, Hercules, CA, USA).

### 2.3. Sequencing of PCR Products and Bioinformatic Analysis

IHHNV and WSSV positive PCR products obtained from crab samples were purified and sequenced using ABI 3730xl DNA analyzer (Eurofins Genomics India Pvt. Ltd., Bengaluru, India). The obtained sequences were analysed for finding homology with other sequences using BLAST program in NCBI database (Bethesda, MD, USA). The phylogenetic tree was constructed for the nonstructural protein 1 (NS1) gene of IHHNV (309 bp) and WSV285 gene of WSSV (570 bp) separately by using MEGA X software version 10.1.7 [44]. The multiple sequence alignment of nucleic acid sequences of the viruses was generated by using the MUSCLE program with default settings. Then ‘Find Best DNA Models’ tool in the MEGA X software version 10.1.7 was used to identify the best-fitting model for constructing a phylogenetic tree. Based on the lowest Bayesian information criterion score, the Kimura 2-parameter model with discrete gamma distribution (K2 + G) and the Tamura 3-parameter (T92) models were identified as the best model for IHHNV and WSSV, respectively. Then the phylogenetic tree was constructed with the maximum likelihood method using the selected models. The neighbour-joining and BioNJ algorithms were used for obtaining the initial tree required for the heuristic search. Missing data and gaps were completely deleted, and the test of phylogeny was done using the bootstrap method with 1000 replications. The online tool interactive tree of life (iTOL, http://itol.embl.de/ accessed on 7 July 2021) was used to visualize the final tree [45].

### 2.4. Histopathological Analysis

Tissue samples were processed for histopathology as per the standard protocol [46]. Finally, the tissue sections (5 µ) were stained by using hematoxylin and eosin.

### 2.5. Analysis of Data

Statistical software SPSS 16.0 was used to calculate the descriptive statistics such as mean and standard error of length (cm) and weight (g) of the collected shrimp and crab samples. The odds ratio is used to measure the strength of association between the exposure and an outcome. The odds ratio values for co-infection of IHHNV and WSSV was computed by using MedCalc statistical software version 20.009 at 95% confidence interval with z statistics and significance level [47].

## 3. Results

### 3.1. Co-Infection of IHHNV and WSSV in Wild Shrimps

A total of 607 shrimp samples comprising of four species were collected from seven fish landing centres located in three districts of Andaman and Nicobar Archipelago (Table 1 and Supplementary Table S1). The PCR analysis revealed that out of 607 shrimp sampled, 123 (20.3%) were found positive for IHHNV alone, 46 (7.6%) were positive for WSSV alone and co-infection of both IHHNV and WSSV was found in 82 (13.5%). Varying degrees of co-infection with IHHNV and WSSV were found across the landing centres and among the shrimp species. Among the districts, the number of shrimp samples with co-infection was higher in South Andaman (n = 52) followed by North and Middle Andaman (n = 20) and Nicobar (n = 10) whereas the positivity rate of co-infection in relation to the total number of shrimp samples collected in that particular district was found higher in Nicobar (40%) followed by South Andaman (18.8%) and North and Middle Andaman (6.5%). Among the shrimp species, co-infection was higher in *Penaeus monodon* (n = 72) followed by *P. merguiensis* (n = 6), *P. indicus* (n = 3) and *P. penicillatus* (n = 1). Within *P. monodon*, the positivity rate of co-infection in relation to the total number of *P. monodon* samples collected in that particular district was found higher in Nicobar (50%) followed by South Andaman (22.7%) and North and Middle Andaman (10.8%). Among the WSSV positive shrimp samples, only 12 samples were found to be positive by first step PCR comprising of *P. monodon* (n = 8), *P. merguiensis* (n = 3) and *P. penicillatus* (n = 1) and the remaining samples were nested PCR positive for WSSV.

### 3.2. Co-Infection of IHHNV and WSSV in Wild Crabs

Altogether, 110 crab samples comprising of four species were collected from a total of eight fish landing centres (Table 2 and Supplementary Table S2). The PCR analysis revealed that out of 110 crabs sampled, 19 (17.3%) were found positive for IHHNV, 9 (8.2%) were positive for WSSV and co-infection of both IHHNV and WSSV was found in 5 (4.5%). Varying degrees of co-infection with IHHNV and WSSV were found across the landing centres and among the crab species. Among the districts, the number of crab samples with co-infection was higher in South Andaman (n = 2) and Nicobar (n = 2) followed by North and Middle Andaman (n= 1) whereas the positivity rate of co-infection in relation to the total number of crab samples collected in that particular district was found higher in Nicobar (11.8%) followed by South Andaman (3.2%) and North and Middle Andaman (3.2%). Among the crab species, co-infection was higher in *S. serrata* (n = 3) followed by *S. tranquebarica* (n = 1) and *P. pelagicus* (n = 1) whereas co-infection was not observed in *P. reticulatus.* On the other hand, *P. reticulatus* was tested positive for IHHNV only. Within *S. serrata*, the positivity rate of co-infection in relation to the total number of *S. serrata* samples collected in that particular district was found higher in Nicobar (10%) followed by North and Middle Andaman (3.2%) and South Andaman (2.6%). All the WSSV positive crab samples were found to be detected only by nested PCR.

### 3.3. Analysis of Odds Ratio

The odds ratio was evaluated in order to ascertain the strength of association between IHHNV and WSSV incidences during the co-infection. In *P. monodon* and *P. merguiensis*, the incidences of IHHNV infection are significantly associated with the incidences of WSSV infection whereas no significant association was found in *P. indicus* and *P. penicillatus* (Table 3). Among all the crab species, no significant association was found between the incidences of IHHNV and WSSV (Table 3). Subsequently, a significant association between the incidences of IHHNV and WSSV was observed in shrimp sampling locations such as Junglighat and Lohabarrack landing centres from South Andaman, Kalighat from North and Middle Andaman and Campbell Bay from Nicobar (Table 4). No significant association was observed in other shrimp sampling locations. Consequently, no significant association between the incidences of IHHNV and WSSV was observed in any of the crab sampling locations (Table 4).

### 3.4. Sequence Analysis of IHHNV and WSSV

**Figure 2:**
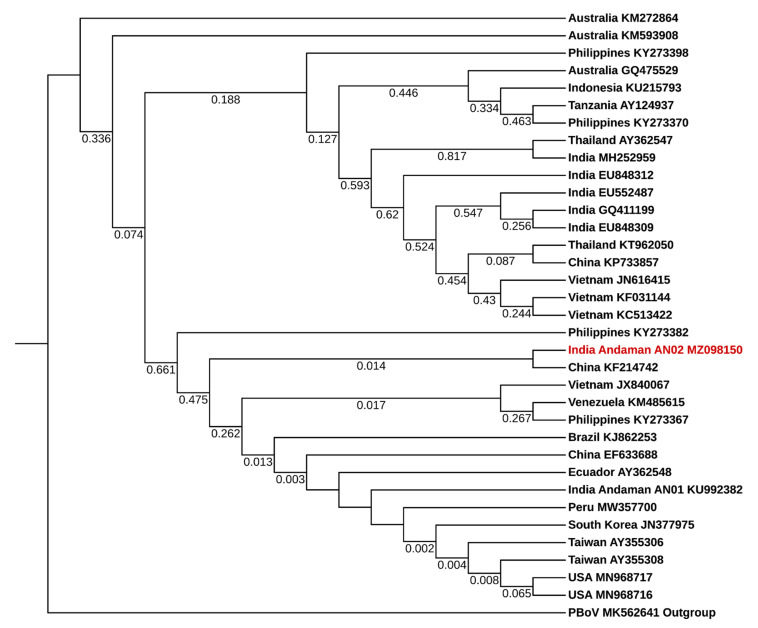
Phylogenetic tree of nonstructural protein 1 (NS1) gene of IHHNV. The Genbank accession numbers are provided along with the country name and the IHHNV sequence obtained in this present study (Genbank accession number MZ098150) was highlighted in red colour text. The tree was constructed using the maximum likelihood method with Kimura 2-parameter model (K2). The NS1 gene of porcine bocavirus (PBoV) was used as an outgroup (Genbank accession number MK562641).

The BLAST analysis of the nucleic acid sequence of IHHNV obtained from crab of Andaman and Nicobar Archipelago (GenBank accession number MZ098150) in the present study revealed 99.68% sequence identity to the sequences of IHHNV reported from Andaman AN-01 (KU992382), China (KU373072), Australia (KM272863), Peru (MW357700), Vietnam (JX840067), Texas (MN968717), Florida (MN968716), Taiwan (AY355308) and Ecuador (AY362548), 99.67% with Philippines (KY273368), 99.35% with Venezuela (KM485615) and Brazil (KJ862253), 99.03% with South Korea (JN377975), 96.44% with India (MH252959), 96.12% with Thailand (AY362547) and 95.78% with Indonesia (KU215793). Phylogenetic analysis showed that the IHHNV sequence obtained from the present study revealed the closest relationship with other IHHNV sequences retrieved from various countries (Figure 2 and Supplementary Table S3). Similarly, the IHHNV sequence derived from the present study has emerged in the same clade as the earlier reported IHHNV sequence (Genbank accession number KU992382) obtained from the wild shrimps of Andaman and Nicobar Islands [41].

**Figure 3:**
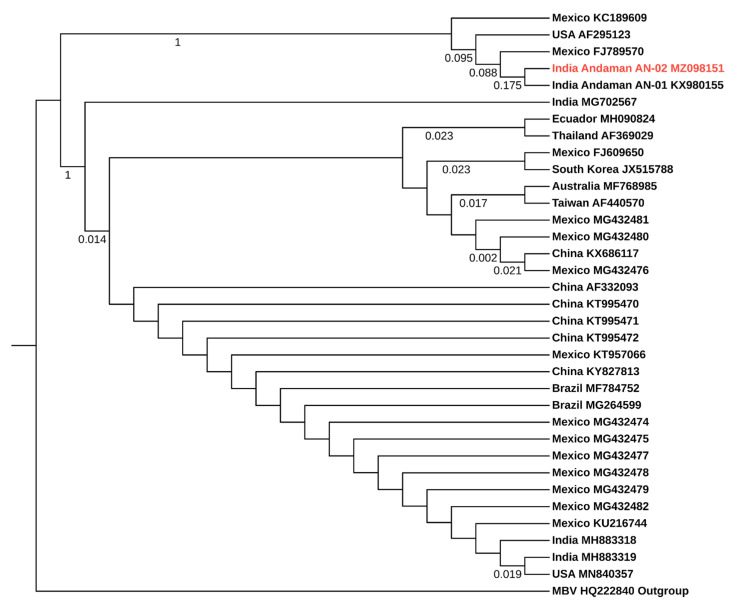
Phylogenetic tree of WSV285 gene of WSSV. The Genbank accession numbers are provided along with the country name and the WSSV sequence obtained in this present study (Genbank accession number MZ098151) was highlighted in red colour text. The tree was constructed using the maximum likelihood method with Tamura 3-parameter model (T92). The polyhedrin-like gene of monodon baculovirus (MBV) was used as an outgroup (Genbank accession number HQ222840).

Similarly, the BLAST analysis of WSSV sequence obtained from *S. serrata* of Andaman and Nicobar Archipelago (GenBank accession number MZ098151) in the present study showed 100% sequence identity with the WSSV sequence reported earlier from wild shrimps of Andaman AN-01 (KX980155), 99.82% with USA (MN840357), Mexico (KU216744), Brazil (MG264599), China (KY827813), South Korea (JX515788), Thailand (AF369029), Taiwan (AF440570) and India (MH883319), 99.65% with Ecuador (MH090824) and Australia (MF768985). Phylogenetic analysis revealed that WSSV obtained from the present study exhibited the closest relationship with WSSV sequences obtained from other countries (Figure 3 and Supplementary Table S4). Moreover, as mentioned earlier, the WSSV sequence obtained from this present study clade with the WSSV sequence (Genbank accession number KX980155) reported earlier from the wild shrimps of Andaman and Nicobar Islands [40].

### 3.5. Histopathology

**Figure 4:**
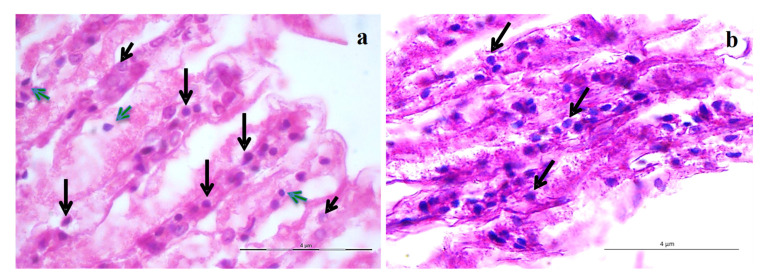
Histopathology of the shrimp (*Penaeus monodon*) gill sections. (**a**). Gill sections showing intra-nuclear eosinophilic cowdry type A inclusion bodies (long black arrow) pathognomonic to IHHNV and eosinophilic to basophilic inclusion bodies suggestive of WSSV infection (short green arrow) and normal cells (short black arrow). (**b**). Gill sections showing WSSV characteristic more basophilic intra-nuclear inclusion bodies (black arrow). Scale bar 4 µm.

Histopathology analysis of the infected animals revealed a severe infection with hypertrophied and pyknotic nuclei with eosinophilic, basophilic and cowdry type A intra-nuclear inclusion bodies. The infected shrimp gill section revealed the eosinophilic intra-nuclear cowdry type A inclusion bodies inside the hypertrophied nucleus pathognomonic to IHHNV and eosinophilic to basophilic intra-nuclear inclusion bodies suggestive of WSSV infection (Figure 4). Further, the infected shrimp gill section showed severe infection of WSSV with characteristics intra-nuclear inclusion bodies inside the hypertrophied nucleus (Figure 4). The WSSV characteristic inclusion bodies were fully developed, more basophilic and granular in texture. No occlusion bodies were observed in the gill sections.

## 4. Discussion

Among the pathogens, WSSV is considered as the most devastating pathogen as it brings mass mortality and crop losses in the crustacean aquaculture sector. The WSSV was first reported in 1992, since then it has emerged as the dreadful pathogen known to infect a majority of the shrimp and crab species in various countries and remains a global challenge to the aquaculture industry [24,48]. Whereas IHHNV may not cause mass mortality but known to incur slow and stunted growth, resulting in severe economic losses [49]. As compared with the research efforts on crustacean viral diseases in the aquaculture sector, limited reports are available on the spread or contamination of viruses in wild crustaceans [26]. Further, the studies on the co-infection of viruses in wild crustaceans are still very limited. The summary on the single and co-infection of various viruses in the wild crustaceans reported from different countries is provided in Table 5. The present study has been instigated in order to understand the level of co-infection of IHHNV and WSSV in wild crustaceans with a focus on shrimps and crabs in the Andaman and Nicobar Archipelago.

Infections of IHHNV and WSSV in the wild crustaceans of Andaman and Nicobar Archipelago have been reported by few researchers with various levels of prevalence [39,40,41]. A study conducted by ICAR-Central Institute of Brackishwater Aquaculture during 2004 in the South Andaman district revealed the presence of WSSV in wild crustaceans such as *P. monodon*, *P. semisulcatus*, *F. merguiensis* and *Portunus pelagicus* by nested PCR (unpublished). WSSV was also detected by either first step or nested PCR in wild-caught *P. monodon*, *F. merguiensis* and *Scylla serrata* samples collected from South and North Andaman districts during the period 2006 to 2007 [39]. In our earlier studies, WSSV was detected in wild *P. monodon* samples collected from South Andaman and Nicobar districts whereas IHHNV was detected in wild *P. monodon* samples collected from South Andaman, North and Middle Andaman, and Nicobar districts during the period 2015 to 2016 [40,41]. However, there is no information available on the co-infection of crustacean viruses, namely, IHHNV and WSSV from Andaman and Nicobar Archipelago. In the present study conducted during 2018 to 2020 by covering all the three districts of Andaman and Nicobar Archipelago, co-infection of IHHNV and WSSV was detected in all the tested shrimp species viz., *P. monodon, P. merguiensis, P. indicus* and *P. penicillatus* whereas in the case of crab, co-infection was observed only in *S. serrata, S. tranquebarica* and *P. pelagicus.* These findings clearly indicate that long-term surveillance with more samples, species and geographical coverage provides the tangible indication of the establishment of co-infection of IHHNV and WSSV in the wild crustaceans of Andaman and Nicobar Archipelago. Similarly, various levels of prevalence of these viruses were recorded elsewhere in wild crustaceans [26,55,56,57,58] and shrimps can also serve as asymptomatic carriers which may remain infected for a long period of time [6,55]. Disease surveillance is a continuous process which requires regular monitoring in order to understand the prevalence of viruses in the wild population which may vary over the period of time based on various factors [50,54,55]. It is noteworthy to mention that in the present study, co-infection of IHHNV and WSSV was found in wild crustaceans of all the three districts of Andaman and Nicobar Archipelago which provides further insights on the spread of these viruses in the wild conditions.

In this present study, the prevalence of IHHNV was higher than that of WSSV in both wild shrimps and crabs. It is further substantiated that the low level of WSSV infections may be due to the season, temperature, co-infection, or pre-infection with IHHNV and host as evidenced from the earlier study [9]. The IHHNV infection mechanism and its virulence varies with different species and developmental stages of the host [59]. Further, the majority of the obtained WSSV infections in wild shrimp and crab species are restricted to nested PCR positive which may be due to the fact that it is hard to find acute infection and dead animals as weak and moribund animals are certainly susceptible prey for various predators in the wild environment [9]. Further, the shrimp and crab samples detected with WSSV by nested PCR did not show any clinical white spots. It has been reported that the host affected with low level of infection may not show any clinical signs and non-penaeid species such as crab mostly have sub-clinical infection in the natural environment [10]. The earlier reports on WSSV infection also substantiates the low level of infection or nested PCR positive as obtained in this present study [39,40]. Co-infection was found higher in *P. monodon* than any other shrimp species which is also validated with the earlier reports in which the infection of WSSV and IHHNV was higher in *P. monodon* [39,40,41] and clearly indicates that these Islands alike Southeast Asian countries are within the geographical range of these viruses [3,17,60]. Natural viral infections with a wide range of prevalence were also observed in wild crabs of the Asian region [13,24,51,61]. On the other hand, the low level of co-infection in wild crabs observed in the present study cannot be overlooked as the crabs may serve as reservoirs to spread these viruses [23,48]. It is reported that both horizontal and vertical transmissions are responsible for these viral disease transmissions in the natural environment [3,55,58,62,63,64] and it is difficult to gauge the impact of these viral diseases in wild crustaceans [54]. The odds ratio analysis revealed that the presence or incidence of IHHNV infection was found to exacerbate the incidence of WSSV infection and vice versa in shrimp species such as *P. monodon* and *P. merguiensis* and also for the sampling locations such as Junglighat, Lohabarrack, Kalighat and Campbell Bay. However, pre-infection with IHHNV was reported to reduce the WSSV incidences in wild crustaceans [9], which warrants further investigation on the biological association between IHHNV and WSSV infection in wild crustaceans.

The high identity between IHHNV and WSSV sequences derived from crab samples in the present study and IHHNV and WSSV sequences reported from wild shrimps of Andaman and Nicobar Islands further confirmed that these particular strains of IHHNV and WSSV are widely prevalent among wild crustaceans of Andaman and Nicobar Archipelago. The IHHNV and WSSV infections were also confirmed by histopathology which further supported the PCR results. Histopathology of the infected shrimp gill tissues revealed the eosinophilic intra-nuclear cowdry type A inclusion bodies confirming the IHHNV infection and eosinophilic to basophilic and more basophilic intra-nuclear inclusion bodies denoting the WSSV infection as reported in earlier studies to support the specific infections [55,65].

It is speculated that the possible establishment of IHHNV and WSSV infections in wild crustaceans of Andaman and Nicobar Archipelago, India might be due to natural oceanic currents or ballast/ bilge water from the ships navigating through the Islands [9,28]. As Andaman and Nicobar Archipelago is located in close proximity to the Southeast Asian countries and sharing the Andaman Sea, there may be possibilities that the infected host or vectors might be carried away by the water currents. This speculation is in line with the previous study, where the alongshore currents from Brazil transported the infectious agents up to 5500 km, and also the fluctuation in current impacts the prevalence of WSSV in Brazil [9,66,67]. Similarly, the ballast/ bilge water from the transporting vessels might have contributed to the transmission of infections through the infected host or vectors. However, further studies are also recommended in order to validate these possibilities to arrive at a definitive conclusion.

## 5. Conclusions

This is the first report on the co-infection of IHHNV and WSSV in wild crustaceans of Andaman and Nicobar Archipelago, India, which provides further information on the extent of the spread of these viruses and biogeography among the wild populations. It is noteworthy to mention that the research on viral infections in wild crustaceans should be given due priority as such viral infections could possibly endanger the potential of brackishwater aquaculture sector in these Islands. Therefore, continuous disease surveillance or monitoring is the need of the hour to gauge the dynamics of the prevalence of pathogens in wild crustaceans and their associated ecosystems. Further, biosecurity measures should be considered as the cardinal sign before venturing into the brackishwater aquaculture sector in Andaman and Nicobar Archipelago towards sustainable aquaculture and ecosystem health management.

## Figures and Tables

**Table 1:** Details of the wild shrimp samples found positive for IHHNV (I), WSSV (W) and Both (B) in Andaman and Nicobar Archipelago. Values within parenthesis indicate the number of samples found positive for IHHNV (I), WSSV (W) and Both (B) by PCR.

District Wise Landing Centres		Number of Samples Collected and Found Positive for IHHNV (I), WSSV (W) and Both (B)				Total Number of Samples and Positive Samples
District	Landing Centre	*P. monodon*	*P. merguiensis*	*P. indicus*	*P. penicillatus*	
South Andaman (SA)	Junglighat	86 (I-17; W-2; B-23)	28 (I-6; W-2; B-4)	26 (I-6; W-3; B-2)	-	140 (I-29; W-7; B-29)
	Lohabarrack	112 (I-19; W-8; B-22)	24 (I-6; W-0; B-1)	-	-	136 (I-25; W-8; B-23)
	Sub-total from SA	198 (I-36; W-10; B-45)	52 (I-12; W-2; B-5)	26 (I-6; W-3; B-2)	-	276 (I-54; W-15; B-52)
North and Middle Andaman (NMA)	Durgapur	51 (I-13; W-6; B-2)	17 (I-2; W-1; B-0)	61 (I-12; W-2; B-0)	-	129 (I-27; W-9; B-2)
	Kalighat	11 (I-2; W-0; B-1)	-	-	20 (I-1; W-1; B-1)	31 (I-3; W-1; B-2)
	Mayabunder	10 (I-4; W-0; B-1)	-	16 (I-2; W-3; B-0)	-	26 (I-6; W-3; B-1)
	Betapur	95 (I-26; W-17; B-14)	25 (I-3; W-0; B-1)	-	-	120 (I-29; W-17; B-15)
	Sub-total from NMA	167 (I-45; W-23; B-18)	42 (I-5; W-1; B-1)	77 (I-14; W-5; B-0)	20 (I-1; W-1; B-1)	306 (I-65; W-30; B-20)
Nicobar (N)	Campbell Bay	18 (I-3; W-1; B-9)	-	7 (I-1; W-0; B-1)	-	25 (I-4; W-1; B-10)
	Sub-total from N	18 (I-3; W-1; B-9)	-	7 (I-1; W-0; B-1)	-	25 (I-4; W-1; B-10)
Grand total number of samples and positive samples from all the districts of Andaman and Nicobar Archipelago		383 (I-84; W-34; B-72)	94 (I-17; W-3; B-6)	110 (I-21; W-8; B-3)	20 (I-1; W-1; B-1)	607 (I-123; W-46; B-82)

**Table 2:** Details of the wild crab samples found positive for IHHNV (I), WSSV (W) and Both (B) in Andaman and Nicobar Archipelago. Values within parenthesis indicate the number of samples found positive for IHHNV (I), WSSV (W) and Both (B) by PCR.

District Wise Landing Centres		Number of Samples Collected and Found Positive for IHHNV (I), WSSV (W) and Both (B)				Total Number of Samples and Positive Samples
District	Landing Centre	*S. serrata*	*S. tranquebarica*	*P. pelagicus*	*P. reticulatus*	
South Andaman (SA)	Guptapara	12 (I-2; W-1; B-0)	7 (I-1; W-1; B-0)	-	-	19 (I-3; W-2; B-0)
	Junglighat	-	-	12 (I-1; W-1; B-1)	4 (I-1; W-0; B-0)	16 (I-2; W-1; B-1)
	Lohabarrack	27 (I-3; W-3; B-1)	-	-	-	27 (I-3; W-3; B-1)
	Sub-total from SA	39 (I-5; W-4; B-1)	7 (I-1; W-1; B-0)	12 (I-1; W-1; B-1)	4 (I-1; W-0; B-0)	62 (I-8; W-6; B-2)
North and Middle Andaman (NMA)	Durgapur	17 (I-4; W-1; B-1)	-	-	-	17 (I-4; W-1; B-1)
	Rangat Bay	8 (I-1; W-0; B-0)	-	-	-	8 (I-1; W-0; B-0)
	Kadamtala	6 (I-2; W-1; B-0)	-	-	-	6 (I-2; W-1; B-0)
	Sub-total from NMA	31 (I-7; W-2; B-1)	-	-	-	31 (I-7; W-2; B-1)
Nicobar (N)	Campbell Bay	10 (I-2; W-1; B-1)	2 (I-1; W-0; B-0)	-	-	12 (I-3; W-1; B-1)
	Car Nicobar	-	5 (I-1; W-0; B-1)	-	-	5 (I-1; W-0; B-1)
	Sub-total from N	10 (I-2; W-1; B-1)	7 (I-2; W-0; B-1)	-	-	17 (I-4; W-1; B-2)
Grand total number of samples and positive samples from all the districts of Andaman and Nicobar Archipelago		80 (I-14; W-7; B-3)	14 (I-3; W-1; B-1)	12 (I-1; W-1; B-1)	4 (I-1; W-0; B-0)	110 (I-19; W-9; B-5)

**Table 3:** Odds ratio values for co-infection of IHHNV and WSSV in different wild shrimp and crab species. The confidence interval was calculated by using MedCalc statistical software. If the 95% confidence interval excludes 1.0, the association is statistically significant at *p* < 0.05. If the 95% confidence interval includes 1.0, the association is not statistically significant at *p* < 0.05.

Shrimp/Crab Species	Odds Ratio	95% Confidence Interval		Z Statistics	*p* Value
		Lower	Upper		
Shrimp species					
*P. monodon*	4.87	3.01	7.88	6.439	0.0001
*P. merguiensis*	8	1.81	35.3	2.746	0.006
*P. indicus*	1.39	0.34	5.71	0.46	0.6455
*P. penicillatus*	17	0.55	523.79	1.62	0.1052
Crab species					
*S. serrata*	1.71	0.39	7.48	0.717	0.4735
*S. tranquebarica*	3	0.14	64.26	0.703	0.4823
*P. pelagicus*	9	0.28	285.52	1.246	0.2129
*P. reticulatus*	2.33	0.029	182.92	0.381	0.7034

**Table 4:** Odds ratio values for co-infection of IHHNV and WSSV in wild shrimp and crab samples collected from various landing centres. The confidence interval was calculated by using MedCalc statistical software. If the 95% confidence interval excludes 1.0, the association is statistically significant at *p* < 0.05. If the 95% confidence interval includes 1.0, the association is not statistically significant at *p* < 0.05.

District Wise Landing Centres	Odds Ratio	95% Confidence Interval		Z Statistics	*p* Value
		Lower	Upper		
Wild shrimps					
South Andaman (SA)					
Junglighat	10.71	4.23	27.16	4.998	0.0001
Lohabarrack	9.2	3.66	23.12	4.721	0.0001
Sub-total from SA	9.95	5.18	19.11	6.9	0.0001
North and Middle Andaman (NMA)					
Durgapur	0.75	0.15	3.68	0.356	0.7218
Kalighat	16.67	1.14	243.72	2.056	0.0398
Mayabunder	0.89	0.08	10.3	0.094	0.9249
Betapur	1.8	0.79	4.09	1.391	0.1642
Sub-total from NMA	1.96	1.04	3.69	2.086	0.037
Nicobar (N)					
Campbell Bay	25	2.36	264.8	2.673	0.0075
Sub-total from N	25	2.36	264.8	2.673	0.0075
Wild crabs					
South Andaman (SA)					
Guptapara	0.83	0.03	21.43	0.113	0.9098
Junglighat	6	0.26	140.05	1.115	0.2649
Lohabarrack	2.22	0.17	28.98	0.609	0.5422
Sub-total from SA	1.92	0.33	11.23	0.721	0.4707
North and Middle Andaman (NMA)					
Durgapur	2.75	0.14	55.17	0.661	0.5085
Rangat Bay	5	0.07	366.35	0.735	0.4626
Kadamtala	0.47	0.01	16.89	0.416	0.6772
Sub-total from NMA	1.5	0.12	19.18	0.312	0.7552
Nicobar (N)					
Campbell Bay	2.33	0.1	50.99	0.538	0.5903
Car Nicobar	7	0.17	291.36	1.023	0.3064
Sub-total from N	5	0.35	71.9	1.183	0.2367

**Table 5:** Summary of single and co-infection of various viruses in the wild crustaceans reported from different countries.

Name of the Crustacean Species	Name of the Viruses as Single/Co-Infection	Country	Year	Reference
*Penaeus monodon*	IHHNV, WSSV, MBV, HPV	Indonesia	2014	[25]
*P. monodon*	IHHNV, WSSV, YHV	Thailand	2012–2013	[26]
*P. monodon*	IHHNV, WSSV, MBV	Philippines	2014–2015	[27]
*Scylla olivacea*, *S. tranquebarica* and *S. paramamosain*	WSSV	Malaysia	2015	[28]
*Euphausia pacifica*, *Leptochela gracilis*, *Latreutes anoplonyx*, *L. planirostris*, *Acetes chinensis*, *Crangon affinis*, *Palaemon graviera*, *Alpheus japonicus*, *A. distinguendus*, *Trachypenaeus curvirostris* and *Penaeus chinensis*	WSSV	China	2016–2018	[50]
*Uca* spp. and *Sesarma* spp. *Scylla serrata*, *Sesarma oceanica*, *Matuta planipes* and *Charybdis lucifera* *Parapenaeopsis stylifera*, *Penaeus monodon*, *P. indicus*, *Metapenaeus dobsoni*, *M. affinis*, *Heterocarpus woodmasoni*, *Scylla serrata*, *S. tranquebarica*, *Portunus sanguinolentus*, *P. pelagicus*, *Charybdis cruciata* and *Panulirus homarus*	IHHNV, WSSV WSSV WSSV	India India India	2014–2015 1999 2015	[24] [51] [52]
*Artemesia longinaris*, *Cyrtograpsus angulatus* and *Palaemon macrodactylus*	IHHNV, WSSV	Argentina	2003–2009	[9]
*Farfantepenaeus paulensis* *Neohelice granulata*	WSSV IHHNV, WSSV	Brazil Brazil	2008 2008	[53] [23]
*Penaeus notialis* and *P. brasiliensis* *P. stylirostris*	IHHNV IHHNV	Mexico Mexico	2016–2017 1996	[54] [55]
*L. vannamei*	IHHNV, WSSV	Panama	2000	[56]

## Data Availability

The sequences of IHHNV and WSSV from this study are available in the NCBI database under the accession numbers MZ098150 and MZ098151, respectively.

## Supplementary Materials

The following are available online at https://www.mdpi.com/article/10.3390/v13071378/s1, Table S1: Length and weight of the shrimp samples collected from Andaman and Nicobar Archipelago, Table S2: Length and weight of the crab samples collected from Andaman and Nicobar Archipelago, Table S3: NCBI accession numbers and IHHNV nucleic acid sequences used for the construction of phylogenetic tree, Table S4: NCBI accession numbers and WSSV nucleic acid sequences used for the construction of phylogenetic tree.
