# Supplementary material for: Co-Infection of Infectious Hypodermal and Hematopoietic Necrosis Virus (IHHNV) and White Spot Syndrome Virus (WSSV) in the Wild Crustaceans of Andaman and Nicobar Archipelago, India
Source: Viruses. 2021 Jul 15;13(7):1378. doi: 10.3390/v13071378 (PMC8310313; doi:10.3390/v13071378)
Supplement: Supplementary file 1 [file viruses-13-01378-s001.zip › viruses-1262061-supplementary.pdf]

Supplementary Table S1: Length and weight of the shrimp samples collected from Andaman and Nicobar Archipelago. The mean length (cm) and mean weight (g) of the shrimp samples were provided as mean ± standard error.

**District wise landing centres**		**Number of samples with mean length (ML) in cm and mean weight (MW) in g**			
**District**	**Landing centre**	***P. monodon***	***P. merguiensis***	***P. indicus***	***P. penicillatus***
South Andaman	Junglighat	86 (ML: 16.2 ± 0.3; MW: 30.1 ± 1.5)	28 (ML: 14.7 ± 0.2; MW: 16.8 ± 0.9)	26 (ML: 14.4 ± 0.4; MW: 18.5 ± 1.1)	–
	Lohabarrack	112 (ML: 20.3 ± 0.3; MW: 63.2 ± 2.8)	24 (ML: 14.2 ± 0.2; MW: 18.9 ± 0.7)	–	–
North and Middle Andaman	Durgapur	51 (ML: 16.7 ± 0.6; MW: 35.3 ± 4.1)	17 (ML: 12.7 ± 0.7; MW: 14.3 ± 0.9)	61 (ML: 13.2 ± 0.4; MW: 15.1 ± 0.6)	–
	Kalighat	11 (ML: 20.0 ± 1.1; MW: 56.7 ± 8.1)	–	–	20 (ML: 13.9 ± 0.2; MW: 14.9 ± 0.8)
	Mayabunder	10 (ML: 18.8 ± 1.0; MW: 48.1 ± 8.0)	–	16 (ML: 14.6 ± 0.3; MW: 17.0 ± 0.8)	–
	Betapur	95 (ML: 22.5 ± 0.8; MW: 87.0 ± 10.8)	25 (ML: 16.0 ± 0.5; MW: 27.1 ± 2.7)	–	–
Nicobar	Campbell Bay	18 (ML: 19.1 ± 0.6; MW: 51.3 ± 3.3)	–	7 (ML: 14.4 ± 0.2; MW: 17.1 ± 1.1)	–

.

Supplementary Table S2: Length and weight of the crab samples collected from Andaman and Nicobar Archipelago. The mean carapace length (cm) and mean weight (g) of the crab samples were provided as mean ± standard error.

**District wise landing centres**		**Number of samples with mean carapace length (ML) in cm and mean weight (MW) in g**			
**District**	**Landing centre**	***S. serrata***	***S. tranquebarica***	***P. pelagicus***	***P. reticulatus***
South Andaman	Guptapara	12 (ML: 5.4 ± 0.2; MW: 98.8 ± 11.8)	7 (ML: 5.8 ± 0.2; MW: 104.7 ± 8.4)	–	–
	Junglighat	–	–	12 (ML: 4.8 ± 0.2; MW: 71.4 ± 6.7)	4 (ML: 5.0 ± 0.3; MW: 77.4 ± 6.3)
	Lohabarrack	27 (ML: 6.7 ± 0.2; MW: 152.7 ± 13.5)	–	–	–
North and Middle Andaman	Durgapur	17 (ML: 6.4 ± 0.2; MW: 129.5 ± 7.8)	–	–	–
	Rangat Bay	8 (ML: 5.9 ± 0.3; MW: 104.4 ± 13.0)	–	–	–
	Kadamtala	6 (ML: 7.1 ± 0.2; MW: 185.6 ± 28.4)	–	–	–
Nicobar	Campbell Bay	10 (ML: 5.5 ± 0.1; MW: 95.3 ± 3.7)	2 (ML: 6.0 ± 0.3; MW: 102.4 ± 12.7)	–	–
	Car Nicobar	–	5 (ML: 8.4 ± 1.0; MW: 350.9 ± 71.2)	–	–

Supplementary Table S3: NCBI accession numbers and IHHNV nucleic acid sequences used for the construction of phylogenetic tree.

>India_Andaman_AN01_KU992382

TCCAACACTTAGTCAAAACCAAGTCAGCAAGAACAGTACAAGAACTTGTCAATAAACTTGACGATGAAGAATATAAACAA

CTATGGACCCGTACCAGAGGACAATATAAAGACAAACTCAGAGGAATATTAACATACTACAACAACAAGAAAAAGTCAAA

CCAAAGCCAACTGTCACTAATTACAAACCTGCAGAATATATCAAAAAGAAAACCAGACTACGACAATATGCAGTGGATAA

AATACATGTTAGCCAACAACGACATCCGTGTACCAGAAATCTTAGCTTGGATAATCATCGTAGCAGACA

>Vietnam_JX840067

TCCAACACTTAGTCAAAACCAAGTCAGCAAGAACAGTACAAGAACTTGTCAATAAACTTGACGATGAAGAATATAAACAA

CTATGGACCCGTACCAGAGGACAATATAAAGACAAACTCAGAGGAATATTAACATACTACAACAACAAGAAAAAGTCAAA

CCAAAGCCAACTGTCACTAATTACAAACCTGCAGAATATATCAAAAAGAAAACCAGACTACGACAATATGCAGTGGATAA

AATACATGTTAGCCAACAACGACATCCGTGTACCAGAAATCTTAGCTTGGATAATCATCGTAGCAGACA

>Taiwan_AY355308

TCCAACACTTAGTCAAAACCAAGTCAGCAAGAACAGTACAAGAACTTGTCAATAAACTTGACGATGAAGAATATAAACAA

CTATGGACCCGTACCAGAGGACAATATAAAGACAAACTCAGAGGAATATTAACATACTACAACAACAAGAAAAAGTCAAA

CCAAAGCCAACTGTCACTAATTACAAACCTGCAGAATATATCAAAAAGAAAACCAGACTACGACAATATGCAGTGGATAA

AATACATGTTAGCCAACAACGACATCCGTGTACCAGAAATCTTAGCTTGGATAATCATCGTAGCAGACA

>South_Korea_JN377975

TCCAACACTTAGTCAAATCCAAGTCAGCAAGAACAGTACAAGAACTTGTCAATAAACTTGACGATGAAGAATATAAACAA

CTATGGACCCGTACCAGAGGACAATATAAAGACAAACTCAGAGGAATATTAACATACTACAACAACAAGAAAAAGTCAAA

CCAAAGCCAACTGTCACTAATTACAAACCTGCAGAATATATCAAAAAGAAAACCAGACTACGACAATATGCAGTGGATAA

AGTACATGTTAGCCAACAACGACATCCGTGTACCAGAAATCTTAGCTTGGATAATCATCGTAGCAGACA

>India_MH252959

TCCAACACTTAGTCAAAACCAAATCTGCAAGAACAGTCCAAGAACTTGTCAATAAACTTGACGATGAAGAATACAAACAA

CTATGGACCCGTACCAGAGGACAATATAAAGACAAACTCAGAGGAATACTAACATACTACAACAACAAGAAAAAGTCAAA

CCAAAGCCAACTGTCACTCATTACAAACCTGCAAAATATATCAAAAAGGAAACCAGACTACGACAACATGCAATGGATAA

AATACATGTTAGCCAACAACGACATCCGTGTACCAGAAATCTTAGCTTGGATAATCATCGTAGCAGACA

>Thailand_AY362547

TCCAACACTTAGTCAAAACCAAATCTGCAAGAACAGTCCAAGAACTTGTCAATAAACTTGACGATGAAGAATACAAACAA

CTATGGACACGTACCAGAGGACAATATAAAGACAAACTCAGAGGAATACTAACATACTACAACAACAAGAAAAAGTCAAA

CCAAAGCCAACTGTCACTCATTACAAACCTGCAAAATATATCAAAAAGGAAACCAGACTACGACAACATGCAATGGATAA

AATACATGTTAGCCAACAACGACATCCGTGTACCAGAAATCTTAGCTTGGATAATCATCGTAGCAGACA

>Indonesia_KU215793

TCCAACACTTAGTCAAAACCAAATCAGCAAGAACAGTACAAGAACTTGTCAATAAACTTGACGATGAAGAATACAAACAA

CTATGGACCCGTACCAGAGGACAATATAAAGACAAACTCAGAGGAATACTGACATACTACAACAACAAGAAAAAGTCAAA

CCAGAGCCAACTGTCACTCATTACAAATCTTCAAAATATATCAAAAAGGAAACCAGACTACGACAACATGCAATGGATAA

AATACATGTTAGCCAACAACGACATCCGTGTACCAGAAATCTTAGCTTGGATAATCATCGTAGCAGAC

>India_EU848312

TCCAACACTTAGTCAAAACCAAATCTGCAAGAACAGTCCAAGAACTTGTCAATAAACTTGACGATGAGGAATACAAACAG

CTATGGACCCGCACCAGAGGACAATATAAAGACAAACTCAGAGGAATACTAACATACTACAACAACAAGAAAAAGTCGAA

CCAAAGCCAACTGTCACTCATTACAAACCTGCAAAATATATCAAAAAGGAAACCAGACTACGACAACATGCAATGGATAA

AATACATGTTAGCCAACAACGACATCCGTGTACCAGAAATCTTAGCTTGGATAATCATCGTAGCAGACA

>Tanzania_AY124937

ACACCTGGTCAAAACCAAGTCAGCAAGAACAGTACAAGAACTTGTCAATAAACTTGACGATGAAGAATACAAACAACTAT

GGACCCGTACTAGAGGACAATATAAAGACAAACTCAGAGGAATACTGACATACTACAACAACAAGAAAAAGTCAAACCAA

AGCCAACTATCACTCATTACAAATCTTCAAAATATTTCAAAAAGGAAACCAGACTACGACAACATGCAATGGATAAAGTA

CATGTTAGCCAACAACGACATCCGTGTACCAGAAATCTTGGCTTGGATCCTTATAGTTGCAGACA

>India_Andaman_AN02_MZ098150

TCCAACACTTAGTCAAAACCAAGTCAGCAAGAACAGTACAAGAACTTGTCAATAAACTTGACGATGAAGAATATAAACAA

CTATGGACCCGTACCAGAGGACAATATAAAGACAAACTCAGAGGAATATTAACATACTACAACAACAAGAAAAAGTCAAA

CCAAAGCCAACTGTCACTAATTACAAACCTGCAGAATATATCAAAAAGAAAACCAGACTACGACAATATGCAGTGGATAA

AATATATGTTAGCCAACAACGACATCCGTGTACCAGAAATCTTAGCTTGGATAATCATCGTAGCAGACA

>PBoV_MK562641_Outgroup

AGGCAGCCGATCACTCACTATCTCAGGAAGGGTCTGCATGACCGTCTGGTCATGAACAGCAGCGAGGATACCGGGGAGCC

GGTACACAAGATGGCTCGCTGGGGAGATCTGCCTCAGGTCAGTGAGAATTCTCTGGCTAGACAGAACACTCAGACCAGAC

CGACTAAAATCAACAAGAAACAGCATCTCATGTTAGATACTCTACAGAGGTGCGAGGAGCAGTTCATCTGCACTAAGGAG

GAGCTGACCATGCTCCATCCTGATGTGGTGATCATGTTCGAGAGCACGCCTAGCGGGTCTCGGACGCTA

>Peru_MW357700

TCCAACACTTAGTCAAAACCAAGTCAGCAAGAACAGTACAAGAACTTGTCAATAAACTTGACGATGAAGAATATAAACAA

CTATGGACCCGTACCAGAGGACAATATAAAGACAAACTCAGAGGAATATTAACATACTACAACAACAAGAAAAAGTCAAA

CCAAAGCCAACTGTCACTAATTACAAACCTGCAGAATATATCAAAAAGAAAACCAGACTACGACAATATGCAGTGGATAA

AATACATGTTAGCCAACAACGACATCCGTGTACCAGAAATCTTAGCTTGGATAATCATCGTAGCAGACA

>USA_MN968717

TCCAACACTTAGTCAAAACCAAGTCAGCAAGAACAGTACAAGAACTTGTCAATAAACTTGACGATGAAGAATATAAACAA

CTATGGACCCGTACCAGAGGACAATATAAAGACAAACTCAGAGGAATATTAACATACTACAACAACAAGAAAAAGTCAAA

CCAAAGCCAACTGTCACTAATTACAAACCTGCAGAATATATCAAAAAGAAAACCAGACTACGACAATATGCAGTGGATAA

AATACATGTTAGCCAACAACGACATCCGTGTACCAGAAATCTTAGCTTGGATAATCATCGTAGCAGACA

>USA_MN968716

TCCAACACTTAGTCAAAACCAAGTCAGCAAGAACAGTACAAGAACTTGTCAATAAACTTGACGATGAAGAATATAAACAA

CTATGGACCCGTACCAGAGGACAATATAAAGACAAACTCAGAGGAATATTAACATACTACAACAACAAGAAAAAGTCAAA

CCAAAGCCAACTGTCACTAATTACAAACCTGCAGAATATATCAAAAAGAAAACCAGACTACGACAATATGCAGTGGATAA

AATACATGTTAGCCAACAACGACATCCGTGTACCAGAAATCTTAGCTTGGATAATCATCGTAGCAGACA

>Ecuador_AY362548

TCCAACACTTAGTCAAAACCAAGTCAGCAAGAACAGTACAAGAACTTGTCAATAAACTTGACGATGAAGAATATAAACAA

CTATGGACCCGTACCAGAGGACAATATAAAGACAAACTCAGAGGAATATTAACATACTACAACAACAAGAAAAAGTCAAA

CCAAAGCCAACTGTCACTAATTACAAACCTGCAGAATATATCAAAAAGAAAACCAGACTACGACAATATGCAGTGGATAA

AATACATGTTAGCCAACAACGACATCCGTGTACCAGAAATCTTAGCTTGGATAATCATCGTAGCAGACA

>Venezuela_KM485615

TCCAACACTTAGTCAAAACCAAGTCAGCAAGAACAGTACAAGAACTTGTCAATAAACTTGACGATGAAGAATATAAACAA

CTATGGACCCGTACCAGAGGACAATATAAAGACAAACTCAGAGGAATATTAACATACTACAACAACAAGAAAAAGTCAAA

CCAAAGCCAACTGTCACTAATTACAAACCTGCAGAATATATCAAAAAGAAAACCAGACTACGACAATATGCAGTGGATAA

AATACATGTTAGCCAACAACGACATCCGTGTACCAGAAATCTTAGCATGGATAATCATCGTAGCAGACA

>Brazil_KJ862253

TCCAACACTTAGTCAAAACCAAGTCAGCAAGAACAGTACAAGAACTTGTCAATAAACTTGACGATGAAGAATATAAACAA

CTATGGACCCGTACCAGAGGACAATATAAAGACAAACTCAGAGGAATATTAACATACTACAACAACAAGAAAAAGTCAAA

CCAAAGCCAACTGTCACTAATTACAAACCTGCAGAATATATCAAAAAGAAAACCAGACTACGACAATATGCAGTGGATAA

AATACATGTTAGCCAACAACAACATCCGTGTACCAGAAATCTTAGCTTGGATAATCATCGTAGCAGACA

>China_EF633688

TCCAACACTTAGTCAAAACCAAGTCAGCAAGAACAGTACAAGAACTTGTCAATAAACTTGACGATGAAGAATATAAACAA

CTATGGACCCGTACCAGAGGACAATATAAAGACAAACTCAGAGGAATATTAACATACTACAACAACAAGAAAAAGTCAAA

CCAAAGCCAACTGTCACTAATTACAAACCTGCAGAATATATCAAAAAGAAAACCAGACTACGACAATATGCAGTGGATAA

AATACATGTTAGCCAACAACGACATCCGTGTACCAGAAATCTTAGCTTGGATAATCATCGTAGCAGACA

>Taiwan_AY355306

TCCAACACTTAGTCAAAACCAAGTCAGCAAGAACAGTACAAGAACTTGTCAATAAACTTGACGATGAAGAATATAAACAA

CTATGGACCCGTACCAGAGGACAATATAAAGACAAACTCAGAGGAATATTAACATACTACAACAACAAGAAAAAGTCAAA

CCAAAGCCAACTGTCACTAATTACAAACCTGCAGAATATATCAAAAAGAAAACCAGACTACGACAATATGCAGTGGATAA

AATACATGTTAGCCAACAACGACATCCGTGTACCAGAAATCTTAGCTTGGATAATCATCGTAGCAGACA

>Philippines_KY273382

CAACACTTAGTCAAAACCAAGTCAGCAAGAACAGTACAAGAACTTGTCAATAAACTTGACGATGAAGAATATAAACAACT

ATGGACCCGTACCAGAGGACAATATAAAGACAAACTCAGAGGAATATTAACATACTACAACAACAAGAAAAAGTCGAACC

AAAGCCAACTGTCACTAATTACAAACCTGCAAAATATATCAAAAAGAAAACCAGACTACGACAATATGCAGTGGATAAAA

TACATGTTAGCCAACAACGACATCCGTGTACCAGAAATCTTAGCTTGGATAATCATCGTAGCAGAC

>China_KF214742

TCCAACACTTAGTCAAAACCAAGTCAGCAAGAACAGTACAAGAACTTGTCAATAAACTTGACGATGAAGAATATAAACAA

CTATGGACCCGTACCAGAGGACAATATAAAGACAAACTCAGAGGAATATTAACATACTACAACAACAAGAAAAAGTCAAA

CCAATGCCAACTGTCACTAATTACAAACCTGCAGAATATATCAAAAAGAAAACCAGACTACGACAATATGCATTGGATAA

AATACATGTTAGCCAACAACAACATCCGTGTACCAGAAATCTTAGCTTGGATAATCATCGTAGCAGACA

>Philippines_KY273367

CAACACTTAGTCAAAACCAAGTCAGCAAGAACAGTACAAGAACTTGTCAATAAACTTGACGATGAAGAATATAAACAACT

ATGGACCCGTACCAGAGGACAATATAAAGACAAACTCAGAGGAATATTAACATACTACAACAACAAGAAAAAGTCAAACC

AAAGCCAACTGTCACTAATTACAAACCTGCAGAATATATCAAAAAGAAAACCAGACTACGACAATATGCAGTGGATAAAA

TACGTGTTAGCCAACAACGACGTCCGTGTACCAGAAATCTTAGCTTGGATAATCATCGGAGCAGAC

>Australia_KM593908

TCCAACACTTAGTCAAAACCAAGTCAGCAAGAACAGTACAAGAACTTGTCAATAAACTTGACGATGAAGAATACAAACAA

CTATGGACCCGTACCAGAGGACAATATAAAGACAAACTCAGAGGAATACTAACATACTACAACAACAAGAAAAAGTCAAA

CCAAAGCCAACTGTCACTCATTACAAACCTGCAAAATATATCAAAAAGGAAACCAGACTACGACAACATGCAGTGGATAA

AATACATGTTAGCCAACAACGACATCCGTGTACCAGAAATCTTAGCTTGGATAATCATCGTAGCAGACA

>Philippines_KY273398

CAACACTTAGTCAAAACCAAATCAGCAAGAACAGTACAAGAACTTGTCAATAAACTTGACGACGAAGAATACAAACAACT

ATGGACCCGTACCAGAGGACAATATAAAGACAAACTCAGAGGAATACTAACATACTACAACAACAAGAAAAAGTCAAACC

AAAGCCAACTGTCACTCATTACAAACCTGCAAAATATATCAAAAAGGAAACCAGACTACGACAACATGCAGTGGATAAAA

TACATGTTAGCCAACAACGACATCCGTGTACCAGAAATCTTAGCTTGGATAATCATCGTAGCAGAC

>Australia_GQ475529

TCCAACACTTAGTCAAAACCAAATCAGCAAGAACAGTTCAAGAACTTGTCAATAAACTTGACGATGAAGAATACAAACAA

CTATGGACCCGTACCAGAGGACAATATAAAGACAAACTCAGAGGAATACTGACATACTACAACAACAAGAAAAAGTCAAA

CCAAAGCCAACTGTCACTCATTACAAATCTTCAAAATATATCAAAGAGGAAACCAGACTACGACAATATGCAGTGGATAA

AATATATGTTAGCCAACAACGACATCCGTGTACCAGAAATCTTAGCTTGGATAATCATCGTAGCAGACA

>Philippines_KY273372

CAACACTTAGTCAAAACCAAGTCAGCAAGAACAGTACAAGAACTTGTCAATAAACTTGACGATGAAGAATATAAACAACT

ATGGACCCGTACCAGAGGACAATATAAAGACAAACTCAGAGGAATATTAACATACTACATCATCAAGAAAAAGTCAAACC

AAAGCCAACGGTCACTAATTACAAACCTGCAGAATATATCAAAAAGAAAACCAGACTACCACGATATGCAGTGGATAAAA

TACATGTTAGCCAACAACGACTTCCGTGTAGGGGAAATCTTAGCTTGGATAATCATCGTAGCGGAC

>China_KP733857

TCCAACACTTAGTCAAAACCAAATCTGCAAGAACAGTCCAAGAACTTGTCAATAAACTTGACGATGAGGAATACAAACAG

CTATGGACCCGCACCAGAGGACAATATAAAGACAAACTCAGGGGAATACTAACTTACTACAACAACAAGAAAAAGTCGAA

CCAAAGCCAACTGTCACTAATTACAAACCTGCAGAATATATCAAAAAGGAAACCAGACTACGACAATATGCAGTGGATAA

AGTACATGTTAGCCAACAACGACATCCGTGTACCAGAAATCTTAGCTTGGATAATCATCGTAGCAGACA

>Thailand_KT962050

TCCAACACTTAGTCAAAACCAAATCTGCAAGAACAGTCCAAGAACTTGTCAATAAACTTGACGATGAGGAATACAAACAG

CTATGGACCCGTACCAGAGGACAATATAAAGACAAACTCAGGGGAATACTAACTTACTACAACAACAAGAAAAAGTCGAA

CCAAAGCCAACTGTCACTCATTACAAACCTGCAAAATATATCAAAAAGGAAACCAGACTACGACAACATGCAGTGGATAA

AGTACATGTTAGCCAACAACGACATCCGTGTACCAGAAATCTTAGCTTGGATAATCATCGTAGCAGACA

>Vietnam_KC513422

TCCAACACTTAGTCAAAACCAAATCTGCAAGAACAGTCCAAGAACTTGTCAATAAACTTGACGATGAGGAATACAAACAG

CTATGGACCCGCACCAGAGGACAATATAAAGACAAACTCAGGGGAATACTAACTTACTACAACAACAAGAAAAAGTCAAA

CCAAAGCCAACTGTCACTCATTACAAACCTGCAAAATATATCAAAAAGGAAACCAGACTACGACAACATGCAGTGGATAA

AGTACATGTTAGCCAACAACGACATCCGTGTACCAGAAATCTTAGCTTGGATAATCATCGTAGCAGACA

>Vietnam_JN616415

TCCAACACTTAGTCAAAACCAAATCTGCAAGAACAGTCCAAGAACTTGTCAATAAACTTGACGATGAGGAATACAAACAG

CTATGGACCCGCACCAGAGGACAATATAAAGACAAACTCAGGGGAATACTAACTTACTACAACAACAAGAAAAAGTCAAA

CCAAAGCCAACTGTCACTCATTACAAACCTGCAAAATATATCAAAAAGGAAACCAGACTACGACAACATGCAGTGGATAA

AGTACATGTTAGCCAACAACGACATCCGTGTACCAGAAATCTTAGCTTGGATAATCATCGTAGCAGACA

>India_GQ411199

TCCAACACTTAGTCAAAACCAAATCTGCAAGAACAGTCCAAGAACTTGTCAATAAACTTGACGATGAGGAATACAAACAG

CTATGGACCCGCACCAGAGGACAATATAAAGACAAACTCAGAGGAATACTAACTTACTACAACAACAAGAAAAAGTCGAA

CCAAAGCCAACTGTCACTCATTACAAACCTGCAAAATATATCAAAAAGGAAACCAGACTACGACAACATGCAGTGGATAA

AGTACATGTTAGCCAACAACGACATCCGTGTACCAGAAATCTTAGCTTGGATAATCATCGTAGCAGACA

>India_EU848309

TCCAACACTTAGTCAAAACCAAATCTGCAAGAACAGTCCAAGAACTTGTCAATAAACTTGACGATGAGGAATACAAACAG

CTATGGACCCGCACCAGAGGACAATATAAAGACAAACTCAGAGGAATACTAACTTACTACAACAACAAGAAAAAGTCGAA

CCAAAGCCAACTGTCACTCATTACAAACCTGCAAAATATATCAAAAAGGAAACCAGACTACGACAACATGCAGTGGATAA

AGTACATGTTAGCCAACAACGACATCCGTGTACCAGAAATCTTAGCTTGGATAATCATCGTAGCAGACA

>India_EU552487

TCCAACACTTAGTCAAAACCAAATCTGCAAGAACAGTCCAAGAACTTGTCAATAAACTTGACGATGAGGAATACAAACAG

CTATGGACCCGCACCAGAGGACAATATAAAGACAAACTCAGAGGAATACTAACTTACTACAACAACAAGAAAAAGTCGAA

CCAAAGCCAACTGTCACTCATTACAAACCTGCAAAATATATCAAAAAGGAAACCAGACTACGACAACATGCAGTGGATAA

AGTACATGTTAGCCAACAACGACATCCGTGTACCAGAAATCTTAGCTTGGATAATCATCGTAGCAGACA

>Australia_KM272864

GTCGGCAAGAACAGTACAAGAACTTGTCAATAAACTTGACGATGAAGAATACAAACAACTATGGACCCGTACCAGAGGAC

AATATAAAGACAAACTCAGAGGAATACTAACATACTACAACAACAAGAAAAAGTCAAACCAAAGCCAACTGTCACTCATT

ACAAACCTGCAAAATATATCAAAAAGGAAACCAGACTACAACAACATGCAGTGGATAAAATACATGTTAGCCAACAACGA

CATCCGTGTACCAGAAATCTTAGCTTGGATAATCATCGTAGCAGACA

>Vietnam_KF031144

TCCAACACTTAGTCAAAACCAAATCTGCAAGAACAGTCCAAGAACTTGTCAATAAACTTGACGATGAGGAATACAAACAG

CTATGGACCCGCACCAGAGGACAATATAAAGACAAACTCAGGGGAATACTAACTTACTACAACAACAAGAAAAAGTCGAA

CCAAAGCCAACTGTCACTCATTACAAACCTGCAAAATATATCAAAAAGGAAACCAGACTACGACAACATGCAGTGGATAA

AGTACATGTTAGCCAACAACGACATCCGTGTACCAGAAATCTTAGCTTGGATAATCATCGTAGCAGACA

>Philippines_KY273370

CAACACTTAGTCAAAACCAAATCAGCAAGAACAGTACAAGAACTCGTCAATAAACTCGACGATGAAGAATACAAACAACT

ATGGACTCGAACAAGAGGACAATATAAAGACAAACTCAGAGGGATACTAACATACTACAACAACAAGAAAAAGTCAAACC

AAAGCCAACTATCCCTGATCACAAATCTTCAGAATATCTCAAAAAGAAAACCAGACTACGATAACATGCAATGGATAAAG

TATATGCTAGCCAACAACGACATCCGTGTACCAGAAATTTTGGCTTGGATAATCATCGTAGCAGAC

Supplementary Table S4: NCBI accession numbers and WSSV nucleic acid sequences used for the construction of phylogenetic tree.

>USA_MN840357

TAACGCTATCCAGTATCACGAATCACTATGCTCTTTGCACTCTAGCATCTCTTATGCAGACTATTATACGTCTCGCAATA

ACAATTCTGAAGATGGAGGAGGAAACTCTTCTTCAGAAAAGAGCAATGCAGATGTAGCCAAGACTATGGCCTCTTTCTAT

GACCAGTTCGATAAGAGTGAAGACAGCAAGAAAAATAAGAACAAAACTTCAAATGAGATCCTTATAAAAATGTTCCAAAT

GGATAGGGTTTTGGATGGCATGGATGATGATGATGATGAAGATAGTGATAGTAGTAGCAGTGAGAATGAAGAGGAGGAGG

AAGAGGAGGAAATTGTAAAGAAACCAGCAAAGAAGAGGAAAGTGGAAGATGTTGATAGCAATAAGAAGACACTGCCAAAG

GAACCTGCCGTTAAGAAGGTGAAGCAGGAAGAAGATGTGGAGATGGAGGAAGTGAAGGAAGCAGCAGCAGAAGAAGAAAA

GAAAGAGGAACAGGAGGCGAAGGAGGAAGACGCTACTGAGTATGACGACGATACAGAAGAGGACGAGAAAGCAGTAGCAT

CTGATGAAGA

>India_MH883319

TAACGCTATCCAGTATCACGAATCACTATGCTCTTTGCACTCTAGCATCTCTTATGCAGACTATTATACGTCTCGCAATA

ACAATTCTGAAGATGGAGGAGGAAACTCTTCTTCAGAAAAGAGCAATGCAGATGTAGCCAAGACTATGGCCTCTTTCTAT

GACCAGTTCGATAAGAGTGAAGACAGCAAGAAAAATAAGAACAAAACTTCAAATGAGATCCTTATAAAAATGTTCCAAAT

GGATAGGGTTTTGGATGGCATGGATGATGATGATGATGAAGATAGTGATAGTAGTAGCAGTGAGAATGAAGAGGAGGAGG

AAGAGGAGGAAATTGTAAAGAAACCAGCAAAGAAGAGGAAAGTGGAAGATGTTGATAGCAATAAGAAGACACTGCCAAAG

GAACCTGCCGTTAAGAAGGTGAAGCAGGAAGAAGATGTGGAGATGGAGGAAGTGAAGGAAGCAGCAGCAGAAGAAGAAAA

GAAAGAGGAACAGGAGGCGAAGGAGGAAGACGCTACTGAGTATGACGACGATACAGAAGAGGACGAGAAAGCAGTAGCAT

CTGATGAAGA

>India_MH883318

TAACGCTATCCAGTATCACGAATCACTATGCTCTTTGCACTCTAGCATCTCTTATGCAGACTATTATACGTCTCGCAATA

ACAATTCTGAAGATGGAGGAGGAAACTCTTCTTCAGAAAAGAGCAATGCAGATGTAGCCAAGACTATGGCCTCTTTCTAT

GACCAGTTCGATAAGAGTGAAGACAGCAAGAAAAATAAGAACAAAACTTCAAATGAGATCCTTATAAAAATGTTCCAAAT

GGATAGGGTTTTGGATGGCATGGATGATGATGATGATGAAGATAGTGATAGTAGTAGCAGTGAGAATGAAGAGGAGGAGG

AAGAGGAGGAAATTGTAAAGAAACCAGCAAAGAAGAGGAAAGTGGAAGATGTTGATAGCAATAAGAAGACACTGCCAAAG

GAACCTGCCGTTAAGAAGGTGAAGCAGGAAGAAGATGTGGAGATGGAGGAAGTGAAGGAAGCAGCAGCAGAAGAAGAAAA

GAAAGAGGAACAGGAGGCGAAGGAGGAAGACGCTACTGAGTATGACGACGATACAGAAGAGGACGAGAAAGCAGTAGCAT

CTGATGAAGA

>Mexico_KU216744

TAACGCTATCCAGTATCACGAATCACTATGCTCTTTGCACTCTAGCATCTCTTATGCAGACTATTATACGTCTCGCAATA

ACAATTCTGAAGATGGAGGAGGAAACTCTTCTTCAGAAAAGAGCAATGCAGATGTAGCCAAGACTATGGCCTCTTTCTAT

GACCAGTTCGATAAGAGTGAAGACAGCAAGAAAAATAAGAACAAAACTTCAAATGAGATCCTTATAAAAATGTTCCAAAT

GGATAGGGTTTTGGATGGCATGGATGATGATGATGATGAAGATAGTGATAGTAGTAGCAGTGAGAATGAAGAGGAGGAGG

AAGAGGAGGAAATTGTAAAGAAACCAGCAAAGAAGAGGAAAGTGGAAGATGTTGATAGCAATAAGAAGACACTGCCAAAG

GAACCTGCCGTTAAGAAGGTGAAGCAGGAAGAAGATGTGGAGATGGAGGAAGTGAAGGAAGCAGCAGCAGAAGAAGAAAA

GAAAGAGGAACAGGAGGCGAAGGAGGAAGACGCTACTGAGTATGACGACGATACAGAAGAGGACGAGAAAGCAGTAGCAT

CTGATGAAGA

>Mexico_MG432482

TAACGCTATCCAGTATCACGAATCACTATGCTCTTTGCACTCTAGCATCTCTTATGCAGACTATTATACGTCTCGCAATA

ACAATTCTGAAGATGGAGGAGGAAACTCTTCTTCAGAAAAGAGCAATGCAGATGTAGCCAAGACTATGGCCTCTTTCTAT

GACCAGTTCGATAAGAGTGAAGACAGCAAGAAAAATAAGAACAAAACTTCAAATGAGATCCTTATAAAAATGTTCCAAAT

GGATAGGGTTTTGGATGGCATGGATGATGATGATGATGAAGATAGTGATAGTAGTAGCAGTGAGAATGAAGAGGAGGAGG

AAGAGGAGGAAATTGTAAAGAAACCAGCAAAGAAGAGGAAAGTGGAAGATGTTGATAGCAATAAGAAGACACTGCCAAAG

GAACCTGCCGTTAAGAAGGTGAAGCAGGAAGAAGATGTGGAGATGGAGGAAGTGAAGGAAGCAGCAGCAGAAGAAGAAAA

GAAAGAGGAACAGGAGGCGAAGGAGGAAGACGCTACTGAGTATGACGACGATACAGAAGAGGACGAGAAAGCAGTAGCAT

CTGATGAAGA

>Mexico_MG432479

TAACGCTATCCAGTATCACGAATCACTATGCTCTTTGCACTCTAGCATCTCTTATGCAGACTATTATACGTCTCGCAATA

ACAATTCTGAAGATGGAGGAGGAAACTCTTCTTCAGAAAAGAGCAATGCAGATGTAGCCAAGACTATGGCCTCTTTCTAT

GACCAGTTCGATAAGAGTGAAGACAGCAAGAAAAATAAGAACAAAACTTCAAATGAGATCCTTATAAAAATGTTCCAAAT

GGATAGGGTTTTGGATGGCATGGATGATGATGATGATGAAGATAGTGATAGTAGTAGCAGTGAGAATGAAGAGGAGGAGG

AAGAGGAGGAAATTGTAAAGAAACCAGCAAAGAAGAGGAAAGTGGAAGATGTTGATAGCAATAAGAAGACACTGCCAAAG

GAACCTGCCGTTAAGAAGGTGAAGCAGGAAGAAGATGTGGAGATGGAGGAAGTGAAGGAAGCAGCAGCAGAAGAAGAAAA

GAAAGAGGAACAGGAGGCGAAGGAGGAAGACGCTACTGAGTATGACGACGATACAGAAGAGGACGAGAAAGCAGTAGCAT

CTGATGAAGA

>Mexico_MG432478

TAACGCTATCCAGTATCACGAATCACTATGCTCTTTGCACTCTAGCATCTCTTATGCAGACTATTATACGTCTCGCAATA

ACAATTCTGAAGATGGAGGAGGAAACTCTTCTTCAGAAAAGAGCAATGCAGATGTAGCCAAGACTATGGCCTCTTTCTAT

GACCAGTTCGATAAGAGTGAAGACAGCAAGAAAAATAAGAACAAAACTTCAAATGAGATCCTTATAAAAATGTTCCAAAT

GGATAGGGTTTTGGATGGCATGGATGATGATGATGATGAAGATAGTGATAGTAGTAGCAGTGAGAATGAAGAGGAGGAGG

AAGAGGAGGAAATTGTAAAGAAACCAGCAAAGAAGAGGAAAGTGGAAGATGTTGATAGCAATAAGAAGACACTGCCAAAG

GAACCTGCCGTTAAGAAGGTGAAGCAGGAAGAAGATGTGGAGATGGAGGAAGTGAAGGAAGCAGCAGCAGAAGAAGAAAA

GAAAGAGGAACAGGAGGCGAAGGAGGAAGACGCTACTGAGTATGACGACGATACAGAAGAGGACGAGAAAGCAGTAGCAT

CTGATGAAGA

>Mexico_MG432477

TAACGCTATCCAGTATCACGAATCACTATGCTCTTTGCACTCTAGCATCTCTTATGCAGACTATTATACGTCTCGCAATA

ACAATTCTGAAGATGGAGGAGGAAACTCTTCTTCAGAAAAGAGCAATGCAGATGTAGCCAAGACTATGGCCTCTTTCTAT

GACCAGTTCGATAAGAGTGAAGACAGCAAGAAAAATAAGAACAAAACTTCAAATGAGATCCTTATAAAAATGTTCCAAAT

GGATAGGGTTTTGGATGGCATGGATGATGATGATGATGAAGATAGTGATAGTAGTAGCAGTGAGAATGAAGAGGAGGAGG

AAGAGGAGGAAATTGTAAAGAAACCAGCAAAGAAGAGGAAAGTGGAAGATGTTGATAGCAATAAGAAGACACTGCCAAAG

GAACCTGCCGTTAAGAAGGTGAAGCAGGAAGAAGATGTGGAGATGGAGGAAGTGAAGGAAGCAGCAGCAGAAGAAGAAAA

GAAAGAGGAACAGGAGGCGAAGGAGGAAGACGCTACTGAGTATGACGACGATACAGAAGAGGACGAGAAAGCAGTAGCAT

CTGATGAAGA

>Mexico_MG432475

TAACGCTATCCAGTATCACGAATCACTATGCTCTTTGCACTCTAGCATCTCTTATGCAGACTATTATACGTCTCGCAATA

ACAATTCTGAAGATGGAGGAGGAAACTCTTCTTCAGAAAAGAGCAATGCAGATGTAGCCAAGACTATGGCCTCTTTCTAT

GACCAGTTCGATAAGAGTGAAGACAGCAAGAAAAATAAGAACAAAACTTCAAATGAGATCCTTATAAAAATGTTCCAAAT

GGATAGGGTTTTGGATGGCATGGATGATGATGATGATGAAGATAGTGATAGTAGTAGCAGTGAGAATGAAGAGGAGGAGG

AAGAGGAGGAAATTGTAAAGAAACCAGCAAAGAAGAGGAAAGTGGAAGATGTTGATAGCAATAAGAAGACACTGCCAAAG

GAACCTGCCGTTAAGAAGGTGAAGCAGGAAGAAGATGTGGAGATGGAGGAAGTGAAGGAAGCAGCAGCAGAAGAAGAAAA

GAAAGAGGAACAGGAGGCGAAGGAGGAAGACGCTACTGAGTATGACGACGATACAGAAGAGGACGAGAAAGCAGTAGCAT

CTGATGAAGA

>Mexico_MG432474

TAACGCTATCCAGTATCACGAATCACTATGCTCTTTGCACTCTAGCATCTCTTATGCAGACTATTATACGTCTCGCAATA

ACAATTCTGAAGATGGAGGAGGAAACTCTTCTTCAGAAAAGAGCAATGCAGATGTAGCCAAGACTATGGCCTCTTTCTAT

GACCAGTTCGATAAGAGTGAAGACAGCAAGAAAAATAAGAACAAAACTTCAAATGAGATCCTTATAAAAATGTTCCAAAT

GGATAGGGTTTTGGATGGCATGGATGATGATGATGATGAAGATAGTGATAGTAGTAGCAGTGAGAATGAAGAGGAGGAGG

AAGAGGAGGAAATTGTAAAGAAACCAGCAAAGAAGAGGAAAGTGGAAGATGTTGATAGCAATAAGAAGACACTGCCAAAG

GAACCTGCCGTTAAGAAGGTGAAGCAGGAAGAAGATGTGGAGATGGAGGAAGTGAAGGAAGCAGCAGCAGAAGAAGAAAA

GAAAGAGGAACAGGAGGCGAAGGAGGAAGACGCTACTGAGTATGACGACGATACAGAAGAGGACGAGAAAGCAGTAGCAT

CTGATGAAGA

>Brazil_MG264599

TAACGCTATCCAGTATCACGAATCACTATGCTCTTTGCACTCTAGCATCTCTTATGCAGACTATTATACGTCTCGCAATA

ACAATTCTGAAGATGGAGGAGGAAACTCTTCTTCAGAAAAGAGCAATGCAGATGTAGCCAAGACTATGGCCTCTTTCTAT

GACCAGTTCGATAAGAGTGAAGACAGCAAGAAAAATAAGAACAAAACTTCAAATGAGATCCTTATAAAAATGTTCCAAAT

GGATAGGGTTTTGGATGGCATGGATGATGATGATGATGAAGATAGTGATAGTAGTAGCAGTGAGAATGAAGAGGAGGAGG

AAGAGGAGGAAATTGTAAAGAAACCAGCAAAGAAGAGGAAAGTGGAAGATGTTGATAGCAATAAGAAGACACTGCCAAAG

GAACCTGCCGTTAAGAAGGTGAAGCAGGAAGAAGATGTGGAGATGGAGGAAGTGAAGGAAGCAGCAGCAGAAGAAGAAAA

GAAAGAGGAACAGGAGGCGAAGGAGGAAGACGCTACTGAGTATGACGACGATACAGAAGAGGACGAGAAAGCAGTAGCAT

CTGATGAAGA

>Brazil_MF784752

TAACGCTATCCAGTATCACGAATCACTATGCTCTTTGCACTCTAGCATCTCTTATGCAGACTATTATACGTCTCGCAATA

ACAATTCTGAAGATGGAGGAGGAAACTCTTCTTCAGAAAAGAGCAATGCAGATGTAGCCAAGACTATGGCCTCTTTCTAT

GACCAGTTCGATAAGAGTGAAGACAGCAAGAAAAATAAGAACAAAACTTCAAATGAGATCCTTATAAAAATGTTCCAAAT

GGATAGGGTTTTGGATGGCATGGATGATGATGATGATGAAGATAGTGATAGTAGTAGCAGTGAGAATGAAGAGGAGGAGG

AAGAGGAGGAAATTGTAAAGAAACCAGCAAAGAAGAGGAAAGTGGAAGATGTTGATAGCAATAAGAAGACACTGCCAAAG

GAACCTGCCGTTAAGAAGGTGAAGCAGGAAGAAGATGTGGAGATGGAGGAAGTGAAGGAAGCAGCAGCAGAAGAAGAAAA

GAAAGAGGAACAGGAGGCGAAGGAGGAAGACGCTACTGAGTATGACGACGATACAGAAGAGGACGAGAAAGCAGTAGCAT

CTGATGAAGA

>China_KY827813

TAACGCTATCCAGTATCACGAATCACTATGCTCTTTGCACTCTAGCATCTCTTATGCAGACTATTATACGTCTCGCAATA

ACAATTCTGAAGATGGAGGAGGAAACTCTTCTTCAGAAAAGAGCAATGCAGATGTAGCCAAGACTATGGCCTCTTTCTAT

GACCAGTTCGATAAGAGTGAAGACAGCAAGAAAAATAAGAACAAAACTTCAAATGAGATCCTTATAAAAATGTTCCAAAT

GGATAGGGTTTTGGATGGCATGGATGATGATGATGATGAAGATAGTGATAGTAGTAGCAGTGAGAATGAAGAGGAGGAGG

AAGAGGAGGAAATTGTAAAGAAACCAGCAAAGAAGAGGAAAGTGGAAGATGTTGATAGCAATAAGAAGACACTGCCAAAG

GAACCTGCCGTTAAGAAGGTGAAGCAGGAAGAAGATGTGGAGATGGAGGAAGTGAAGGAAGCAGCAGCAGAAGAAGAAAA

GAAAGAGGAACAGGAGGCGAAGGAGGAAGACGCTACTGAGTATGACGACGATACAGAAGAGGACGAGAAAGCAGTAGCAT

CTGATGAAGA

>Mexico_KT957066

TAACGCTATCCAGTATCACGAATCACTATGCTCTTTGCACTCTAGCATCTCTTATGCAGACTATTATACGTCTCGCAATA

ACAATTCTGAAGATGGAGGAGGAAACTCTTCTTCAGAAAAGAGCAATGCAGATGTAGCCAAGACTATGGCCTCTTTCTAT

GACCAGTTCGATAAGAGTGAAGACAGCAAGAAAAATAAGAACAAAACTTCAAATGAGATCCTTATAAAAATGTTCCAAAT

GGATAGGGTTTTGGATGGCATGGATGATGATGATGATGAAGATAGTGATAGTAGTAGCAGTGAGAATGAAGAGGAGGAGG

AAGAGGAGGAAATTGTAAAGAAACCAGCAAAGAAGAGGAAAGTGGAAGATGTTGATAGCAATAAGAAGACACTGCCAAAG

GAACCTGCCGTTAAGAAGGTGAAGCAGGAAGAAGATGTGGAGATGGAGGAAGTGAAGGAAGCAGCAGCAGAAGAAGAAAA

GAAAGAGGAACAGGAGGCGAAGGAGGAAGACGCTACTGAGTATGACGACGATACAGAAGAGGACGAGAAAGCAGTAGCAT

CTGATGAAGA

>China_KT995472

TAACGCTATCCAGTATCACGAATCACTATGCTCTTTGCACTCTAGCATCTCTTATGCAGACTATTATACGTCTCGCAATA

ACAATTCTGAAGATGGAGGAGGAAACTCTTCTTCAGAAAAGAGCAATGCAGATGTAGCCAAGACTATGGCCTCTTTCTAT

GACCAGTTCGATAAGAGTGAAGACAGCAAGAAAAATAAGAACAAAACTTCAAATGAGATCCTTATAAAAATGTTCCAAAT

GGATAGGGTTTTGGATGGCATGGATGATGATGATGATGAAGATAGTGATAGTAGTAGCAGTGAGAATGAAGAGGAGGAGG

AAGAGGAGGAAATTGTAAAGAAACCAGCAAAGAAGAGGAAAGTGGAAGATGTTGATAGCAATAAGAAGACACTGCCAAAG

GAACCTGCCGTTAAGAAGGTGAAGCAGGAAGAAGATGTGGAGATGGAGGAAGTGAAGGAAGCAGCAGCAGAAGAAGAAAA

GAAAGAGGAACAGGAGGCGAAGGAGGAAGACGCTACTGAGTATGACGACGATACAGAAGAGGACGAGAAAGCAGTAGCAT

CTGATGAAGA

>China_KT995471

TAACGCTATCCAGTATCACGAATCACTATGCTCTTTGCACTCTAGCATCTCTTATGCAGACTATTATACGTCTCGCAATA

ACAATTCTGAAGATGGAGGAGGAAACTCTTCTTCAGAAAAGAGCAATGCAGATGTAGCCAAGACTATGGCCTCTTTCTAT

GACCAGTTCGATAAGAGTGAAGACAGCAAGAAAAATAAGAACAAAACTTCAAATGAGATCCTTATAAAAATGTTCCAAAT

GGATAGGGTTTTGGATGGCATGGATGATGATGATGATGAAGATAGTGATAGTAGTAGCAGTGAGAATGAAGAGGAGGAGG

AAGAGGAGGAAATTGTAAAGAAACCAGCAAAGAAGAGGAAAGTGGAAGATGTTGATAGCAATAAGAAGACACTGCCAAAG

GAACCTGCCGTTAAGAAGGTGAAGCAGGAAGAAGATGTGGAGATGGAGGAAGTGAAGGAAGCAGCAGCAGAAGAAGAAAA

GAAAGAGGAACAGGAGGCGAAGGAGGAAGACGCTACTGAGTATGACGACGATACAGAAGAGGACGAGAAAGCAGTAGCAT

CTGATGAAGA

>China_KT995470

TAACGCTATCCAGTATCACGAATCACTATGCTCTTTGCACTCTAGCATCTCTTATGCAGACTATTATACGTCTCGCAATA

ACAATTCTGAAGATGGAGGAGGAAACTCTTCTTCAGAAAAGAGCAATGCAGATGTAGCCAAGACTATGGCCTCTTTCTAT

GACCAGTTCGATAAGAGTGAAGACAGCAAGAAAAATAAGAACAAAACTTCAAATGAGATCCTTATAAAAATGTTCCAAAT

GGATAGGGTTTTGGATGGCATGGATGATGATGATGATGAAGATAGTGATAGTAGTAGCAGTGAGAATGAAGAGGAGGAGG

AAGAGGAGGAAATTGTAAAGAAACCAGCAAAGAAGAGGAAAGTGGAAGATGTTGATAGCAATAAGAAGACACTGCCAAAG

GAACCTGCCGTTAAGAAGGTGAAGCAGGAAGAAGATGTGGAGATGGAGGAAGTGAAGGAAGCAGCAGCAGAAGAAGAAAA

GAAAGAGGAACAGGAGGCGAAGGAGGAAGACGCTACTGAGTATGACGACGATACAGAAGAGGACGAGAAAGCAGTAGCAT

CTGATGAAGA

>China_AF332093

TAACGCTATCCAGTATCACGAATCACTATGCTCTTTGCACTCTAGCATCTCTTATGCAGACTATTATACGTCTCGCAATA

ACAATTCTGAAGATGGAGGAGGAAACTCTTCTTCAGAAAAGAGCAATGCAGATGTAGCCAAGACTATGGCCTCTTTCTAT

GACCAGTTCGATAAGAGTGAAGACAGCAAGAAAAATAAGAACAAAACTTCAAATGAGATCCTTATAAAAATGTTCCAAAT

GGATAGGGTTTTGGATGGCATGGATGATGATGATGATGAAGATAGTGATAGTAGTAGCAGTGAGAATGAAGAGGAGGAGG

AAGAGGAGGAAATTGTAAAGAAACCAGCAAAGAAGAGGAAAGTGGAAGATGTTGATAGCAATAAGAAGACACTGCCAAAG

GAACCTGCCGTTAAGAAGGTGAAGCAGGAAGAAGATGTGGAGATGGAGGAAGTGAAGGAAGCAGCAGCAGAAGAAGAAAA

GAAAGAGGAACAGGAGGCGAAGGAGGAAGACGCTACTGAGTATGACGACGATACAGAAGAGGACGAGAAAGCAGTAGCAT

CTGATGAAGA

>Mexico_KC189609

TCTTCATCAGATGCTACTGCTTTCTCGTCCTCTTCTGTATCGTCGTCATACTCAGTAGCGTCTTCCTCCTTCGCCTCCTG

TTCCTCTTTCTTTTCTTCTTCTGCTGCTGCTTCCTTCACTTCCTCCATCTCCACATCTTCTTCCTGCTTCACCTTCTTAA

CGGCAGGTTCCTTTGGCAGTGTCTTCTTATTGCTATCAACATCTTCCACTTTCCTCTTCTTTGCTGGTTTCTTTACAATT

TCCTCCTCTTCCTCCTCCTCTTCATTCTCACTGCTACTACTATCACTATCTTCATCATCATCATCATCCATGCCATCCAA

AACCCTATCCATTTGGAACATTTTTATAAGGATCTCATTTGAAGTTTTGTTCTTATTTTTCTTGCTGTCTTCACTCTTAT

CGAACTGGTCATAGAAAGAGGCCATAGTCTTGGCTACATCTGCATTGCTCTTTTCTGAAGAAGAGTTTCCTCCTCCATCT

TCAGAATTGTTATTGCGAGACGTATAATAGTCTGCATAAGAGATGCTAGAGTGCAAAGAGCATAGTGATTCGTGATACTG

GATAGCGTTA

>South_Korea_JX515788

TAACGCTATCCAGTATCACGAATCACTATGCTCTTTGCACTCTAGCATCTCTTATGCAGACTATTATACGTCTCGCAATA

ACAATTCTGAAGATGGAGGAGGAAACTCTTCTTCAGAAAAGAGCAATGCAGATGTAGCCAAGACTATGGCCTCTTTCTAT

GACCAGTTCGATAAGAGTGAAGACAGCAAGAAAAATAAGAACAAAACTTCAAATGAGATCCTTATAAAAATGTTCCAAAT

GGATAGGGTTTTGGATGGCATGGATGATGATGATGATGAAGATAGTGATAGTAGTAGCAGTGAGAATGAAGAGGAGGAGG

AAGAGGAGGAAATTGTAAAGAAACCAGCAAAGAAGAGGAAAGTGGAAGATGTTGATAGCAATAAGAAGACACTGCCAAAG

GAACCTGCCGTTAAGAAGGTGAAGCAGGAAGAAGATGTGGAGATGGAGGAAGTGAAGGAAGCAGCAGCAGAAGAAGAAAA

GAAAGAGGAACAGGAGGCGAAGGAGGAAGACGCTACTGAGTATGACGACGATACAGAAGAGGACGAGAAAGCAGTAGCAT

CTGATGAAGA

>Mexico_FJ609650

TAACGCTATCCAGTATCACGAATCACTATGCTCTTTGCACTCTAGCATCTCTTATGCAGACTATTATACGTCTCGCAATA

ACAATTCTGAAGATGGAGGAGGAAACTCTTCTTCAGAAAAGAGCAATGCAGATGTAGCCAAGACTATGGCCTCTTTCTAT

GACCAGTTCGATAAGAGTGAAGACAGCAAGAAAAATAAGAACAAAACTTCAAATGAGATCCTTATAAAAATGTTCCAAAT

GGATAGGGTTTTGGATGGCATGGATGATGATGATGATGAAGATAGTGATAGTAGTAGCAGTGAGAATGAAGAGGAGGAGG

AAGAGGAGGAAATTGTAAAGAAACCAGCAAAGAAGAGGAAAGTGGAAGATGTTGATAGCAATAAGAAGACACTGCCAAAG

GAACCTGCCGTTAAGAAGGTGAAGCAGGAAGAAGATGTGGAGATGGAGGAAGTGAAGGAAGCAGCAGCAGAAGAAGAAAA

GAAAGAGGAACAGGAGGCGAAGGAGGAAGACGCTACTGAGTATGACGACGATACAGAAGAGGACGAGAAAGCAGTAGCAT

CTGATGAAGA

>Thailand_AF369029

TAACGCTATCCAGTATCACGAATCACTATGCTCTTTGCACTCTAGCATCTCTTATGCAGACTATTATACGTCTCGCAATA

ACAATTCTGAAGATGGAGGAGGAAACTCTTCTTCAGAAAAGAGCAATGCAGATGTAGCCAAGACTATGGCCTCTTTCTAT

GACCAGTTCGATAAGAGTGAAGACAGCAAGAAAAATAAGAACAAAACTTCAAATGAGATCCTTATAAAAATGTTCCAAAT

GGATAGGGTTTTGGATGGCATGGATGATGATGATGATGAAGATAGTGATAGTAGTAGCAGTGAGAATGAAGAGGAGGAGG

AAGAGGAGGAAATTGTAAAGAAACCAGCAAAGAAGAGGAAAGTGGAAGATGTTGATAGCAATAAGAAGACACTGCCAAAG

GAACCTGCCGTTAAGAAGGTGAAGCAGGAAGAAGATGTGGAGATGGAGGAAGTGAAGGAAGCAGCAGCAGAAGAAGAAAA

GAAAGAGGAACAGGAGGCGAAGGAGGAAGACGCTACTGAGTATGACGACGATACAGAAGAGGACGAGAAAGCAGTAGCAT

CTGATGAAGA

>USA_AF295123

TCTTCATCAGATGCTACTGCTTTCTCGTCCTCTTCTGTATCGTCGTCATACTCAGTAGCGTCTTCCTCCTTCGCCTCCTG

TTCCTCTTTCTTTTCTTCTTCTGCTGCTGCTTCCTTCACTTCCTCCATCTCCACATCTTCTTCCTGCTTCACCTTCTTAA

CGGCAGGTTCCTTTGGCAGTGTCTTCTTATTGCTATCAACATCTTCCACTTTCCTCTTCTTTGCTGGTTTCTTTACAATT

TCCTCCTCTTCCTCCTCCTCTTCATTCTCACTGCTACTACTATCACTATCTTCATCATCATCATCATCCATGCCATCCAA

AACCCTATCCATTTGGAACATTTTTATAAGGATCTCATTTGAAGTTTTGTTCTTATTTTTCTTGCTGTCTTCACTCTTAT

CGAACTGGTCATAGAAAGAGGCCATAGTCTTGGCTACATCTGCATTGCTCTTTTCTGAAGAAGAGTTTCCTCCTCCATCT

TCAGAATTGTTATTGCGAGACGTATAATAGTCTGCATAAGAGATGCTAGAGTGCAAAGAGCATAGTGATTCGTGATACTG

GATAGCGTTA

>Taiwan_AF440570

TAACGCTATCCAGTATCACGAATCACTATGCTCTTTGCACTCTAGCATCTCTTATGCAGACTATTATACGTCTCGCAATA

ACAATTCTGAAGATGGAGGAGGAAACTCTTCTTCAGAAAAGAGCAATGCAGATGTAGCCAAGACTATGGCCTCTTTCTAT

GACCAGTTCGATAAGAGTGAAGACAGCAAGAAAAATAAGAACAAAACTTCAAATGAGATCCTTATAAAAATGTTCCAAAT

GGATAGGGTTTTGGATGGCATGGATGATGATGATGATGAAGATAGTGATAGTAGTAGCAGTGAGAATGAAGAGGAGGAGG

AAGAGGAGGAAATTGTAAAGAAACCAGCAAAGAAGAGGAAAGTGGAAGATGTTGATAGCAATAAGAAGACACTGCCAAAG

GAACCTGCCGTTAAGAAGGTGAAGCAGGAAGAAGATGTGGAGATGGAGGAAGTGAAGGAAGCAGCAGCAGAAGAAGAAAA

GAAAGAGGAACAGGAGGCGAAGGAGGAAGACGCTACTGAGTATGACGACGATACAGAAGAGGACGAGAAAGCAGTAGCAT

CTGATGAAGA

>Ecuador_MH090824

TAACGCTATCCAGTATCACGAATCACTATGCTCTTTGCACTCTAGCATCTCTTATGCAGACTATTATACGTCTCGCAATA

ACAATTCTGAAGATGGGGGAGGAAACTCTTCTTCAGAAAAGAGCAATGCAGATGTAGCCAAGACTATGGCCTCTTTCTAT

GACCAGTTCGATAAGAGTGAAGACAGCAAGAAAAATAAGAACAAAACTTCAAATGAGATCCTTATAAAAATGTTCCAAAT

GGATAGGGTTTTGGATGGCATGGATGATGATGATGATGAAGATAGTGATAGTAGTAGCAGTGAGAATGAAGAGGAGGAGG

AAGAGGAGGAAATTGTAAAGAAACCAGCAAAGAAGAGGAAAGTGGAAGATGTTGATAGCAATAAGAAGACACTGCCAAAG

GAACCTGCCGTTAAGAAGGTGAAGCAGGAAGAAGATGTGGAGATGGAGGAAGTGAAGGAAGCAGCAGCAGAAGAAGAAAA

GAAAGAGGAACAGGAGGCGAAGGAGGAAGACGCTACTGAGTATGACGACGATACAGAAGAGGACGAGAAAGCAGTAGCAT

CTGATGAAGA

>Australia_MF768985

TAACGCTATCCAGTATCACGAATCACTATGCTCTTTGCACTCTAGCATCTCTTATGCAGACTATTATACGTCTCGCAATA

ACAATTCTGAAGATGGAGGAGGAAACTCTTCTTCAGAAAAGAGCAATGCAGATGTAGCCAAGACTATGGCCTCTTTCTAT

GACCAGTTCGATAAGAGTGAAGACAGCAAGAAAAATAAGAACAAAACTTCAAATGAGATCCTTATAAAAATGTTCCAAAT

GGATAGGGTTTTGGATGGCATGGATGATGATGATGATGAAGATAGTGATAGTAGTAGCAGTGAGAATGAAGAGGAGGAGG

AAGAGGAGGAAATTGTAAAGAAACCAGCAAAGAAGAGGAAAGTGGAAGATGTTGATAGCAATAAGAAGACACTGCCAAAG

GAACCTGCCGTTAAGAAGGTGAAGCAGGAAGAAGATGTGAAGATGGAGGAAGTGAAGGAAGCAGCAGCAGAAGAAGAAAA

GAAAGAGGAACAGGAGGCGAAGGAGGAAGACGCTACTGAGTATGACGACGATACAGAAGAGGACGAGAAAGCAGTAGCAT

CTGATGAAGA

>Mexico_MG432481

TAACGCTATCCAGTATCACGAATCACTATGCTCTTTGCACTCTAGCATCTCTTATGCAGACTATTATACGTCTCGCAATA

ACAATTCTGAAGATGGAGGAGGAAACTCTTCTTCAGAAAAGAGCAATGCAGATGTAGCCAAGACTATGGCCTCTTTCTAT

GACCAGTTCGATAAGAGTGAAGACAGCAAGAAAAATAAGAACAAAACTTCAAATGAGATCCTTATAAAAATGTTCCAAAT

GGATAGGGTTTTGGATGGCATGGATGATGATGATGATGATGAAGATAGTGATAGTAGTAGCAGTGAGAATGAAGAGGAGG

AGGAAGAGGAGGAAATTGTAAAGAAACCAGCAAAGAAGAGGAAAGTGGAAGATGTTGATAGCAATAAGAAGACACTGCCA

AAGGAACCTGCCGTTAAGAAGGTGAAGCAGGAAGAAGATGTGGAGATGGAGGAAGTGAAGGAAGCAGCAGCAGAAGAAGA

AAAGAAAGAGGAACAGGAGGCGAAGGAGGAAGACGCTACTGAGTATGACGACGATACAGAAGAGGACGAGAAAGCAGTAG

CATCTGATGAAGA

>Mexico_MG432480

TAACGCTATCCAGTATCACGAATCACTATGCTCTTTGCACTCTAGCATCTCTTATGCAGACTATTATACGTCTCGCAATA

ACAATTCTGAAGATGGAGGAGGAAACTCTTCTTCAGAAAAGAGCAATGCAGATGTAGCCAAGACTATGGCCTCTTTCTAT

GACCAGTTCGATAAGAGTGAAGACAGCAAGAAAAATAAGAACAAAACTTCAAATGAGATCCTTATAAAAATGTTCCAAAT

GGATAGGGTTTTGGATGGCATGGATGATGATGATGATGATGAAGATAGTGATAGTAGTAGCAGTGAGAATGAAGAGGAGG

AGGAAGAGGAGGAAATTGTAAAGAAACCAGCAAAGAAGAGGAAAGTGGAAGATGTTGATAGCAATAAGAAGACACTGCCA

AAGGAACCTGCCGTTAAGAAGGTGAAGCAGGAAGAAGATGTGGAGATGGAGGAAGTGAAGGAAGCAGCAGCAGAAGAAGA

AAAGAAAGAGGAACAGGAGGCGAAGGAGGAAGACGCTACTGAGTATGACGACGATACAGAAGAGGACGAGAAAGCAGTAG

CATCTGATGAAGA

>Mexico_MG432476

TAACGCTATCCAGTATCACGAATCACTATGCTCTTTGCACTCTAGCATCTCTTATGCAGACTATTATACGTCTCGCAATA

ACAATTCTGAAGATGGAGGAGGAAACTCTTCTTCAGAAAAGAGCAATGCAGATGTAGCCAAGACTATGGCCTCTTTCTAT

GACCAGTTCGATAAGAGTGAAGACAGCAAGAAAAATAAGAACAAAACTTCAAATGAGATCCTTATAAAAATGTTCCAAAT

GGATAGGGTTTTGGATGGCATGGATGATGATGATGATGATGAAGATAGTGATAGTAGTAGCAGTGAGAATGAAGAGGAGG

AGGAAGAGGAGGAAATTGTAAAGAAACCAGCAAAGAAGAGGAAAGTGGAAGATGTTGATAGCAATAAGAAGACACTGCCA

AAGGAACCTGCCGTTAAGAAGGTGAAGCAGGAAGAAGATGTGGAGATGGAGGAAGTGAAGGAAGCAGCAGCAGAAGAAGA

AAAGAAAGAGGAACAGGAGGCGAAGGAGGAAGACGCTACTGAGTATGACGACGATACAGAAGAGGACGAGAAAGCAGTAG

CATCTGATGAAGA

>China_KX686117

TAACGCTATCCAGTATCACGAATCACTATGCTCTTTGCACTCTAGCATCTCTTATGCAGACTATTATACGTCTCGCAATA

ACAATTCTGAAGATGGAGGAGGAAACTCTTCTTCAGAAAAGAGCAATGCAGATGTAGCCAAGACTATGGCCTCTTTCTAT

GACCAGTTCGATAAGAGTGAAGACAGCAAGAAAAATAAGAACAAAACTTCAAATGAGATCCTTATAAAAATGTTCCAAAT

GGATAGGGTTTTGGATGGCATGGATGATGATGATGAAGATAGTGATAGTAGTAGCAGTGAGAATGAAGAGGAGGAGGAAG

AGGAGGAAATTGTAAAGAAACCAGCAAAGAAGAGGAAAGTGGAAGATGTTGATAGCAATAAGAAGACACTGCCAAAGGAA

CCTGCCGTTAAGAAGGTGAAGCAGGAAGAAGATGTGGAGATGGAGGAAGTGAAGGAAGCAGCAGCAGAAGAAGAAAAGAA

AGAGGAACAGGAGGCGAAGGAGGAAGACGCTACTGAGTATGACGACGATACAGAAGAGGACGAGAAAGCAGTAGCATCTG

ATGAAGA

>Mexico_FJ789570

TCTTCATCAGATGCTACTGCTTTCTCGTCCTCTTCTGTATCGTCGTCATACTCACTAGCGTCTTCCTCCTTCGCCTCCTG

TTCCTCTTTCTTTTCTTCTTCTGCTGCTGCTTCCTTCACTTCCTCCATCTCCACATCTTCTTCCTGCTTCACCTTCTTAA

CGGCAGGTTCCTTGGCAGTGTCTTCTTATTGCTATCAACATCTTCCACTTTCCTCTTCTTGCTGGTTTCTTTACAATTTC

CTCCTCTTCCTCCTCCTCTTCATTCTCACTGCTACTACTATCACTATCTTCATCATCATCATCATCCATGCCATCCAAAA

CCCTATCCATTTGGAACATTTTTATAAGGATCTCATTTGAAGTTTTGTTCTTATTTTTCTTGCTGTCTTCACTCTTATCG

AACTGGTCATAGAAAGAGGCCATAGTCTTGGCTACATCTGCATTGCTCTTTTCTGAAGAAGAGTTTCCTCCTCCATCTTC

AGAATTGTTATTGCGAGACGTATAATAGTCTGCATAAGAGATGCTAGAGTGCAAGAGCATAGTGATTCGTGATACTGGAT

AGCGTTA

>India_MG702567

TAACGCTATCCAGTATCACGAATCACTATGCTCTTTGCACTCTAGCATCTCTTATGCAGACTATTATACGTCTCTAACAA

TTCTGAAGATGGAGGAGGAAACTCTTCTTCAGAAAAAGAATGCAGATGTAGCCAAGACTATGGCCTCTTTCTATGACCAG

TCTGATAAGAGTGAAGACAGCAAGAAAAATAAGAACAAACTTCAAATGAGATCCTTATAAAATGTTCCAAATGGATAGGG

TTTTGATGGCATGATGATGATGAAGATAGTGATAGTAGTAGCAGTGAGAATGAAGAGGAGGAGGAAGAGGAGGAAATTGT

AAAGAAACCAGCAAAGAAGAGGAAAGTGGAAGATGTTGATAGCAATAAGAAGACACTGCCAAAGGAACCTGCCGTTAAGA

AGGTGAAGCAGGAAGAAGATGTGAGATGGAGGAAGTGAATGAAGCAGCAGCAGAAGAAGAAAAGAAAGAGGAACAGGAGG

CGAAGGAGGAAGACGCTACTGAGTATGACGACGATACAGAAGAGGACGAGAAAGCAGTAGCATCTGATGAAGA

>India_Andaman_AN01_KX980155

ATGCTACTGCTTTCTCGTCCTCTTCTGTATCGTCGTCATACTCAGTAGCGTCTTCCTCCTTCGCCTCCTGTTCCTCTTTC

TTTTCTTCTTCTGCTGCTGCTTCCTTCACTTCCTCCATCTCCACATCTTCTTCCTGCTTCACCTTCTTAACGGCAGGTTC

CTTTGGCAGTGTCTTCTTATTGCTATCAACATCTTCCACTTTCCTCTTCTTTGCTGGTTTCTTTACAATTTCCTCCTCTT

CCTCCTCCTCTTCATTCTCACTGCTACTACTATCACTATCTTCATCATCATCATCATCCATGCCATCCAAAACCCTATCC

ATTTGGAACATTTTTATAAGGATCTCATTTGAAGTTTTGTTCTTATTTTTCTTGCTGTCTTCACTCTTATCGAACTGGTC

ATAGAAAGAGGCCATAGTCTTGGCTACATCTGCATTGCTCTTTTCTGAAGAAGAGTTTCCTCCTCCATCTTCAGAATTGT

TATTGCGAGACGTATAATAGTCTGCATAAGAGATGCTAGAGTGCAAAGAGCATAGTGATTCGTGATACTGGA

>India_Andaman_AN02_MZ098151

TCTTCATCAGATGCTACTGCTTTCTCGTCCTCTTCTGTATCGTCGTCATACTCAGTAGCGTCTTCCTCCTTCGCCTCCTG

TTCCTCTTTCTTTTCTTCTTCTGCTGCTGCTTCCTTCACTTCCTCCATCTCCACATCTTCTTCCTGCTTCACCTTCTTAA

CGGCAGGTTCCTTTGGCAGTGTCTTCTTATTGCTATCAACATCTTCCACTTTCCTCTTCTTTGCTGGTTTCTTTACAATT

TCCTCCTCTTCCTCCTCCTCTTCATTCTCACTGCTACTACTATCACTATCTTCATCATCATCATCATCCATGCCATCCAA

AACCCTATCCATTTGGAACATTTTTATAAGGATCTCATTTGAAGTTTTGTTCTTATTTTTCTTGCTGTCTTCACTCTTAT

CGAACTGGTCATAGAAAGAGGCCATAGTCTTGGCTACATCTGCATTGCTCTTTTCTGAAGAAGAGTTTCCTCCTCCATCT

TCAGAATTGTTATTGCGAGACGTATAATAGTCTGCATAAGAGATGCTAGAGTGCAAAGAGCATAGTGATTCGTGATACTG

GATAGCCTTA

>MBV_HQ222840_Outgroup

TTATGGATATTTTGCATAAGACATACTTGTAAAGTTGCAAATTTAGGCATTTATTTTTTTCTTAAATTTCACGATTTATA

TATCTAAGTCATGTTCAACGATAATATGATGGATGATGTGAAGCATCACCTCGGTGACCATAAGATGATGCTCACACTTG

CTGCTGCTGGTGCTGCGGCGGGAGCATCATCGATGATGAATGACGCTGCTGACCACCTGAAAAATTATAACGATACTCCT

CCCCAAGAATATTTCAAAAAAGAGAATTCTTCCGGAAAAAGCGAAAGATCATTGATGATGAATTCTTACTTCTCCCTACT

ATGATTGAACCACTGGATCCTCTTGACCTTCCCTAGCCCCATTCACCAAATCATCCTAAAGAAATGCCACATCCTGCGTA

AGTCCTCGTATGGCCAAACTGCTTGCTAAGATTATGAACTATCAGACACTCCATTACTTATTTACCCGCTTTCATACCAA

CCGACGCTTTCTCACTTCCTCCATCTAAAAGCGAATTTATCTCACTTCTTCTATTTCATGAGCTTTTCGGGTCATAGTGA

TGTTTCTTCC
